# The Social Impacts of Circular Strategies in the Apparel Value Chain; a Comparative Study Between Three Countries

**DOI:** 10.1007/s43615-022-00203-8

**Published:** 2022-09-06

**Authors:** Lis J. Suarez-Visbal, Jesús Rosales Carreón, Blanca Corona, Ernst Worrell

**Affiliations:** Copernicus Institute of Sustainable Development, Utrecht University, Vening Meinesz Building A, Princetonlaan 8a, 3584 CB Utrecht, the Netherlands

**Keywords:** Circular economy, Social impact, Circular fashion, Circular strategies, Circular textiles

## Abstract

The apparel value chain is essential for the livelihood of millions of workers around the globe. However, human rights violations and the lack of a sustained income by apparel workers demonstrate the poor working conditions present in this sector. Circular economy (CE) has been used by incumbent businesses and startups as a framework to achieve sustainability, thus contributing to its economic, environmental and social dimensions. However, there is a lack of knowledge on its social impact. Most of the literature assesses CE’s social impacts by focusing only on the number of jobs created. However, the majority of studies agree on the need to analyse further the quality and inclusivity aspects. This paper explores the social impact of the different circular strategies implemented in three countries. It assesses social impacts related to the quality of jobs, workers’ sustainable livelihood and gender equality and inclusion. Results corroborate that CE social ambition is low, and that current circular strategies follow the same feminisation and precariousness of working conditions found in the linear apparel value chain. Thus, policymakers and businesses alike need to strengthen their CE social ambition; coordinate policy and strategies with different countries stakeholders of the apparel value chain to minimise trade-offs; and safeguard a just circular transition. This research contributes to the body of literature on CE by introducing a social impact assessment framework for circularity called SIAF-CE⚥. Additionally, it provides evidence on the current CE social impact implemented by startups and incumbents in regional and global contexts.

**Supplementary Information:**

The online version contains supplementary material available at 10.1007/s43615-022-00203-8.

## Introduction

The apparel value chain (AVC) is essential for the livelihood of millions of workers in several (developing) countries, employing more than 9% of the global working population [1]. Human rights violations, tragedies like the Rana Plaza in Bangladesh [2, 3] and the lack of a sustained income by apparel workers (e.g. during the COVID-19 pandemic) demonstrated the poor working conditions present in this sector. This has also revealed the increasing inequality of social schemes between different regions [4, 5]. Additionally, the apparel sector is considered one of the most polluting ones [2, 6, 7]. Circular economy (CE) has been used by incumbent businesses and startups in the industry as a new framework to achieve sustainability, thus contributing to its economic, environmental and social dimensions [8, 9]. However, there is a lack of knowledge on the social benefits that the CE can provide [10–13].

Most of the literature assesses CE’s social impacts by focusing on the number of jobs created [10–12, 14]. These jobs are developed through different circular strategies (CSs) such as the use of recovered material, rental, repair, resale, remanufacturing and recycling. Even though there is evidence of potential job generation through these CSs, most studies agree on the need to analyse their quality further, the impact on the community, gender equality and inclusion to redress possible harmful effects and trade-offs [15–20]. As CE close performing loops by connecting downstream and upstream stakeholders of the AVC, it can also create a lock-in situation where working conditions become hard to change [21, 22]. Additionally, given the high level of toxic chemicals used in the production, disposal and recovery of textile waste, the implementation of CSs poses specific challenges related to the wellbeing and health of workers and surrounding communities [6, 12, 23]. Finally, CSs in the AVC are labour-intensive and highly feminised and have a high presence of vulnerable populations (e.g. ethnic minorities, refugees and low-skilled low-income workers) [2, 14, 24–27]. Therefore, assessing these distinct aspects of CE in the sector is vital, as circular jobs could recreate the same prevalent low existing working conditions as in the linear business model. This could amplify existing gender, and social inequality gaps should circularity continue to grow in the sector.

Although “Small and medium businesses (SME) in the AVC account for more than 60% of employment in high-income countries, and 50% in low-income countries” [5, 28], most research on CE and CSs implementation has focused on the role of large and established firms [8]. There is scant knowledge about how circular startups and other SMEs can create social impact and better jobs. Furthermore, research on CE and its job creation potential has been concentrated mainly on Europe with few scientific contributions from the global south [9, 23, 29–36]. This paper addresses the lack of knowledge about the social impact of the different CSs implemented in the sector. It will assess the social impacts of CSs in terms of quality of job (QOJ), workers’ sustainable livelihood (SL) and gender equality and inclusion (GE&I) using a novel social impact assessment framework for circularity called *SIAF-CE*⚥ [37]. In addition, the following questions are addressed in this paper. (I) How do the regional and the global AVC compare with each other from the lens of *SIAF-CE*⚥? *This question is relevant because a* comparison between countries that operate in the AVC allows a us to assess how deploying circular strategies in different geographies with a certain degree of proximity (global or local–regional level) influences the quality of jobs, community wellbeing, gender equality and social inclusion, which is also relevant when planning for future territorial development policies*.*

(II) How does the implementation of circular strategies by startups and incumbent companies contrast with each other in terms of social impacts? *This question is relevant because the comparison between incumbent and startup companies allows to assess whether different types of businesses are more drivers than others on creating positive social impact.* Finally, (III) how do different CSs in the apparel sector affect male and female workers?

This research contributes to the body of literature on CE by justifying the use of the *SIAF-CE*⚥ at the micro-level (companies) while providing evidence on the current CSs social impact implemented by startups and incumbents in regional and global contexts. This paper uses a mixed-method approach, and it is organised as follows: the “Theory” section covers the theoretical backbone regarding CE and its social impact ambition. The “Methods” section describes the different methods adopted. The “Results” section contains the results on the social impact of CSs implemented by different types of businesses in the three countries of interest. In the “Discussion” section, the discussion, recommendations for businesses and policymakers, limitations and potential directions for future research are addressed, followed by conclusions in the “Conclusions” section.

## Theory

### Circular Economy in the Apparel Value Chain

The concept of CE has become popular in academia policymakers and businesses due to its capacity to engage a multitude of stakeholders given its systemic approach [9, 38]. However, the current focus of CE remains largely on value creation through better management of material resources but overlooks the social aspects of production processes and parallel socio-cultural transformations [9, 11, 12]. CE can be defined as an economic system that privileges strategies to reduce, reuse, recycle and recover materials in value chain processes, operating at the micro-, meso- and macro-levels, to accomplish sustainable development, creating environmental quality, economic prosperity and social equity, to the benefit of current and future generations [9].

This research focuses on the micro-level of the AVC, which is characterised as a complex system of large and small businesses operating at various geographical locations between the textile, apparel, distribution, retail and textile recycling industries [5, 39]. The AVC is highly feminised, with more than 75% of workers being women [2, 26]. It also holds a high level of job informality, in the production and end-of-life segment [40]. Additionally, it is highly fragmented, which comes with a complex net of relations and power dynamics between the different stakeholders from brands, suppliers, workers etc. [41]. The AVC has historically developed with characteristics that are still very present today. “In the first half of the twentieth century, industrial work in textile and apparel assembly jobs provided an entry point for participation of rural women in the formal economy in developed and developing countries. By the 1970, textile manufacturers had consolidated several global assembly lines that relied on a wide world decentralised and feminised labour force” (pg72, [24]). This phenomenon exploded in the 1990s, catapulting most brands, motivated by cheaper labour and fewer stringent regulations, to migrate their manufacturing facilities to developing nations while keeping the most value-added part of their operation (marketing & sales) in developed countries [42]. This type of production commonly known as fast fashion is characterised by low prices, high collection rotation and massive cheap production mainly done offshore, using a global VC [2, 43, 44]. This *massive* globalisation of production appeared due to technological changes, the evolution of production costs, the emergence of critical international competitors and the elimination of import quotas after 2004 [45].

Additionally, in the AVC, startups and SMEs are not only abundant, but they also play a role in the transition to circularity [8]. Today, companies implement different circular business models (CBM) and circular strategies to extend the useful life of resources, while preserving value at the highest possible level of utility [46, 47]. The R framework has been used to conceptualise the principles of circular economy. It distinguishes between different strategies to embrace circularity in a hierarchical order [48]–[51]. References around the R framework in literature are numerous, ranging from 3 Rs (reduce, reuse, recycle) [52, 53] to 10 Rs (refuse, rethink, reduce, reuse, repair, refurbish, remanufacture, repurpose, recycle, recovery) [9].

There are specific CSs that are most relevant for the AVC. Table 1 describes the CSs used by companies in the AVC compared to the 9R framework used in CE at large. The 9R ladder privileges strategies higher in the R hierarchy, closer to R0. However, in the AVC, these strategies are scant. Table 1 also indicates the place of CSs in the AVC and delineates their territoriality performance. According to [54], circular strategies such as resale, rental, repair and remanufacture are considered most effective on local and regional levels as they capitalise on product proximity while recycling and use of recycled material can be more effective on a regional or at a global scale. This is due to cost consideration, the volume needed for cost-effective recycling and the existence of recycling infrastructure. Additionally, the current recycling process is mainly mechanical, therefore, labour intensive, which motivates companies to offshore this part of the process to cheaper labour countries. To be cost-effective, mechanical recycling requires a larger volume of textile, which is often not enough at the city or local level. Finally, other than territoriality, CSs can be influenced by the ecosystem actors’ interaction, by targeted CE policy and the level of social ambition [54–57].

**Table 1:** Circular strategies applied in the AVC compared with generic CE strategies

PBL 9R	Circular strategies	Global localisation of apparel value chain	Territory	Extraction	Design	Manufacturing	Distribution	Use	End-of-life
Ladder		Description/stage of AVC							
R0 Refuse	Re-design (R1)	Designing apparel with a lifecycle mindset by using DfD (design for disassembly) or DfEoL (design for end of life) [58, 59]	Local/regional		●	●			
R1 Rethink									
R2 Reduce	Reduce/resource Recovery (R2)	Includes on-demand production and incorporation of circular supplies such as recycled yarn, close loop dyes etc. [58, 60]			●	●			
R3 Reuse	Rental (R3)	A product life extension strategy. Refers to paying a fee for using a garment. It includes luxury, well-known brands, local designer and selected vintage items [6, 58, 61, 62]	Local/regional				●	●	
	Resale (R4)	A product life extension strategy. It includes second-hand and vintage items sold online or on brick-and-mortar store [6, 58, 61, 62]					●	●	
R4 Repair	Repair (R5)	A product life extension strategy. It includes onsite in-house, repair tours, third-party repair and DIY kits [58, 61, 63]					●	●	
R5 Refurbished	Remanufacture (R6)	Using parts of a discarded product to create a product with same function [64]. Also include the so-called upcycling fashion understood as “clothing constructed by using reclaimed fabrics, which can either be post-industrial or post-consumer waste and were the quality of the remanufactured fashion clothing is equal or better than brand new fashion clothing” [58, 61, 63, 65]	Regional			●	●	●	
R6 Remanufacture									
R7 Repurpose									
R8 Recycle	Recycle (R7)	Includes all the process from the collection of textiles to sort to actual recycle. It can be mechanical or chemical (the latter one still very new and it is linked directly to the resource recovery or circular supplies) [58, 61, 63]	Regional/global						●
R9 Recover (energy)	Recover (R8)	Once final sorting is done, the scrap left over is then used as feedstock for energy recovery [66]							●

### Social Impacts in AVC Jobs 

The AVC plays a vital role in many countries’ economic development, being an important source of employment creation [67–69]. However, the linear AVC presents high risks of exploitative situations. Annex 1 summarises the most relevant social impacts, specifically in the manufacturing, retail and end-of-life segments. It shows work precariousness with low payment, low working conditions and low workers representation with a high presence of part-time jobs and unconventional working contracts [70–73].

There are several considerations of social impacts in literature; however, CE social impact ambition has been defined by many authors as the creation of jobs [10, 11, 13, 74]. This definition is narrow in scope and depth [16–18, 20]. Even though employment is one of the most critical enablers of poverty reduction, low-quality jobs keep workers disfavoured; quality jobs should also be good for the worker’s families and the communities where they are implemented [19, 75, 76]. Circular strategies are realised through circular jobs. Core circular jobs include repair, resale, rental and recycling [14] while transport logistics, governmental and educational activities have been defined as enablers of secondary jobs [16, 77].

There have been a growing number of studies analysing the potential of a CE to generate jobs. Some reports indicate a positive correlation between CE and employment [16, 20, 75]. Other studies state that increased recycling, reuse, repair and remanufacturing can create jobs for employees displaced from traditional manufacturing and lower structural unemployment [15, 18]. In the AVC, there is scant knowledge over the quantity and quality aspects of these jobs. N. Papú Carrone et al. [77] emphasise the job creation potential of repair and resale in the Dutch AVC, while [34] highlights the recycle job creation potential in Europe and the adverse employment impacts in production countries. Finally, [14] argue that reuse and repair are associated with low salaries and high rates of unpaid work in Europe. This evidences the need for a more detailed social-economic analysis of CSs in the different geographies to avoid employment trade-offs throughout the AVC.

### Social Impact Assessment of Circular Economy

Although several frameworks exist to assess social impacts within the sustainability field, there is scant literature around social impact assessment frameworks (SIAF) for circularity [10, 78, 79]. CE presents system characteristics that can benefit from a more comprehensive social impact assessment approach. This paper uses one of the first framework attempts to cover this gap. This social impact assessment framework is called the SIAF*-CE*⚥ [37]. It takes a worker’s perspective to address critical issues in the AVC, such as gender inequality, inclusiveness and just transition [13, 19, 20, 80]. This framework was operationalised, validated and tested in a previous study with fifty cases in the Netherlands.

**Fig. 1:**
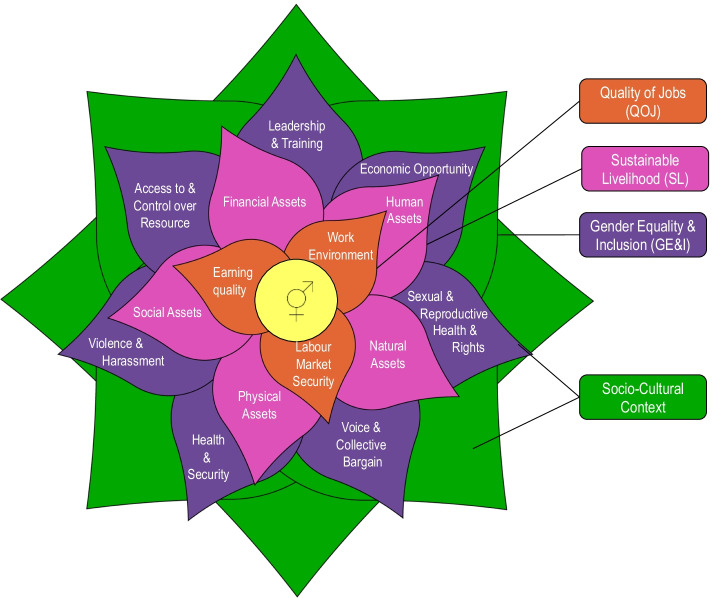
The flower SIAF-CE⚥. Each layer of petals represents one social dimension, and each petal represents an indicator. Outside layers represent the socio-cultural context and power dynamics. Source [37]

As depicted in Fig. 1, the *SIAF-CE*⚥ uses the allegory of a flower where each set of leaves represents three social dimensions: quality of jobs (QOJ), in orange; wellbeing and sustainable livelihoods (SL) in pink leaves; and gender equality and inclusion (GE&I) in purple. The outer leaves in green represent the socio-cultural background and inherent power dynamics. They are not a dimension per se, but they have an overall effect on all other dimensions [81].

The first two dimensions of the *SIAF-CE*⚥ build upon two pre-existing well-known tools, the OECD quality of job framework [82] and the sustainable livelihood framework [83]. The third dimension, GE&I, is based on the eight building blocks of women economic empowerment [84] and in the BSR gender equality social auditing tool [85].

The *SIAF-CE*⚥ measures 15 composite, multi-attribute, qualitative indicators. The quality of job dimension (QOJ) comprises three indicators: *earning quality*, *labour security* and *quality of work environment*. This is the first dimension analysed because it focuses on the job and its direct impact on the individual worker. Wellbeing is represented by the five indicators of the sustainable livelihood framework (*physical assets*, *social assets*, *human assets*, *natural assets and financial assets*). The gender equality and inclusivity (GE&I) dimension considers seven indicators (*economic opportunity*, *training and leadership skills*; *access and control over economic resources and opportunities*; *voice and collective bargain* society; *violence and harassment*; *health and security*; *sexual and reproductive health and rights (SRHR)*)*.* The *SIAF-CE*⚥ uses this multidimensional gender-disaggregated approach to minimise overlooking potential trade-offs among different types of workers, CSs and countries where CSs are employed.

## Methods

### Research Design 

The research was conducted in two phases. The first phase was guided by a qualitative approach consisting of desk research, semi-structured interviews and a thematic and intersectionality analysis. The second phase undertook a quantitative approach consisting of worker surveys based on the *SIAF-CE*⚥*.* Each of the research questions was answered in the “Results” section following the two phases described below.

#### Qualitative Phase

Phase one consisted of three steps: (1) the scoping of the population and sampling selection; (2) the data collection; and (3) the data analysis.

##### Population

The population consisted of businesses that complied with the following criteria: (i) currently implementing one or more of the 5 CSs mentioned in Table 1; (ii) being either a startup^1^ or an incumbent business operating in either the Netherlands, Spain or India; and (iii) having direct staff or subcontracted staff in one of the three locations. These countries were selected because the Netherlands plays a pioneering role in circularity in the AVC. Spain is an important supplier of (sustainable and recycled) fibres, fabrics and clothing to the rest of Europe. At the same time, India is a critical hub for both textile and clothing manufacturing for Europe. Additionally, to select countries, the following criteria were used. Country should (i) play a relevant role in the AVC either as production or consumption countries; (ii) represent geographical diversity; and (iii) have a certain level of implementation of circular economy in the sector and CE policy ambition. Additionally, previous links in these countries facilitated companies’ participation in the research (convenient sampling).

Population includes suppliers of recycled fabrics, manufacturers, brands, retailers and B2C platforms, independent or in-store repair workshops and textile-sorting and recycling companies as well as labour NGOs, academics, think tanks and governmental agencies (considered as experts). Two incumbents and two startups were chosen per country for each of the five CSs.

For interviews, snowball sampling was used. Considering that the AVC comprises diverse stakeholders varying in size, type and role, we classified this group as heterogeneous. Following [86] and [87], saturation was reached for the sample with around 30 interviews in each country, out of which five were with experts and twenty-five with business stakeholders.

##### Data Collection

There were three means of data collection: (i) desk research, (ii) semi-structured interviews and (iii) worker surveys. The desk research was conducted in Google Scholar and Scopus. It included both scientific papers and grey literature from governmental and companies reports and studies from 2000 until 2021. The search terms “social impact of circular strategies”, “circular fashion and social impacts” and “social impacts in the AVC” were used to find relevant scientific literature. The literature review was validated with data triangulation [88, 89].

A total of ninety semi-structured interviews were performed, in all three countries. Experts’ interviews contributed to setting the context of circularity in the sector, and business stakeholders’ interviews contributed to the state of CSs in their own company and their current assessment of social impacts. We interviewed the CSR manager and human resource/operational manager in incumbent firms and the director/founder on startups in each company. Two interview guides were developed based on *SIAF-CE*⚥: one for companies and one for experts and policymakers. Semi-structured interviews were pre-tested and validated with three professionals from the field. As they were anonymous, an identifier was created for each interviewee, starting by country, followed by a C for consolidated, S for startup and an E for expert. Surveys based on the *SIAF-CE*⚥ were composed of 85 multiple-choice and 5 open-ended questions, where 28 referred to socio-demographic factors, 27 to the GE&I dimension, 19 to SL and 16 to QOJ. In [37] operationalisation process, surveys in each country were tested on reliability through a Cronbach’s alpha analysis.

##### Data Analysis

Literature was analysed through content analysis. Each CS by country was considered, emphasising on (i) CE policy for the sector and social impact ambition, (ii) ecosystem development, (iii) the most advanced CSs, along with their territorial focus (local, regional, global) and (iv) their social impact in terms of QOJ, SL and GE&I. To create the ecosystem maps, the 5R framework was used [90]. This framework highlights five basic components of a social system: resources, roles (stakeholders), relations, rules and regulations and results. The data for the map was extracted from the semi-structured interviews and desk research by looking at the place, role and type of relationship between different stakeholders in each country and the AVC. The size of the business bubble was determined based on the type of company (small bubble for startups, medium size bubble for SMEs and larger bubble for consolidated).

Semi-structured interviews were conducted in English, Spanish and Hindi. These were transcribed and translated to English using the software otter.ai. All interviews were coded thematically in three iterations conducted by two other analysts in India and Spain. Pre-selected codes were based on the interview guides for both business and experts and the three dimensions of the social impact assessment framework for circularity *SIAF-CE*⚥. Finally, an additional intersectionality analysis was performed to identify better how the livelihood of the different type of workers is impacted by the different circular jobs created.^2^ Annex 2 shows details on intersectionality.

#### Quantitative Phase

The quantitative phase followed same steps as above.

##### Population

The population was composed of female and male workers directly and indirectly involved in implementing the CSs of startups and incumbents’ businesses identified in the previous phase. An equal representation of male and female workers among the identified CSs was sought. Given the population’s heterogeneity and considering the 15 measurable variables (indicators), the sample size was established at 150, with a minimum sample of 50 subjects for each country [91]. Our final sample consisted of 210 surveys performed in the three countries between January and July 2021 (see Annex 3 for questionnaire).

##### Data Collection

Data in this phase was collected using face-to-face and online surveys based on the SIAF-CE⚥. Surveys were anonymous and confidential and were translated to Dutch, Spanish, Arabic and Hindi (due to the high immigrant proportion of the population). Surveys were conducted in English and these alternative languages by a team of three research analysts that received gender-sensitive training on survey techniques, worker interview approach, building report and addressing sensitive issues.

##### Data Analysis 

The SIAF-CE⚥ responses were ranked on a 4-point Likert scale, ranging between 1 and 4 to score indicators for each dimension, as indicated in Table 2. As several Likert scales were used for different indicators, data were treated as interval data, allowing for descriptive statistics. Frequency, mode and mean were developed. Surveys provided a combination of nominal data and ordinal data. All data sets were verified to be within a range of 1 to 4. During the survey, participants were consistently asked to verify their degree of answer between agreeing and strongly agree and disagree and strongly disagree to suffice controversies around the nuance of responses of Likert scales.

**Table 2:** Scale adopted to rank the indicators of the *SIAF-CE*⚥

Four-point Likert scale scores	General ranking scale for SIAF-CE indicators	Ranking scale for earning quality (QoJ area)
Low (1)	Insufficient/poor performance of the indicator	Below or equal to the poverty line
Medium–low (2)	Sufficient but minimal performance of the indicator	Between the poverty line and minimum wage
Medium–high (3)	Sufficient performance	Between minimum wage and living wage
High (4)	Better than sufficient performance of the indicator	Above living wage and average salary in the sector

Data were included in a Microsoft Excel database. Three different selections were made. First, CSs were grouped by CS, country and workers and then compared with each other. Then it was grouped by male and female workers and by business typology and then compared. Finally, CSs were grouped by CSs, country and workers and then compared.

Results were presented in a circular bar chart showing the value of the indicators from (1) *low* to (4) *high*. The graph is divided equally into three areas to plot the indicators of each dimension (*QOJ*, *SL* and *GE&I*). Each indicator is shown with two bars, one bar for females and another for male workers [37].

## Results

Results are organised by research question: first question by country, second question by business type and third question by circular strategies.

### Regional and Global Apparel Value Chain Social Impacts

In this section, an argumentation of each country national CE policy and social impact ambition is made, followed by a discussion about ecosystem development and most developed circular strategies. Then, the social impacts of the AVC in the three countries are compared with the *SIAF-CE*⚥ framework.

#### The Netherlands

The Netherlands has a national ambition for CE and a specific policy regarding the AVC. This is summarised in the “Policy program for Circular textile 2020–2025”. The current social ambition is only linked to creating jobs and paying a living wage [92, 93]. For businesses, the social ambition is linked to creating local or regional jobs by reshoring the value chain back to Europe (NLS10, NLS01, NLS05, NLE02).

**Fig. 2:**
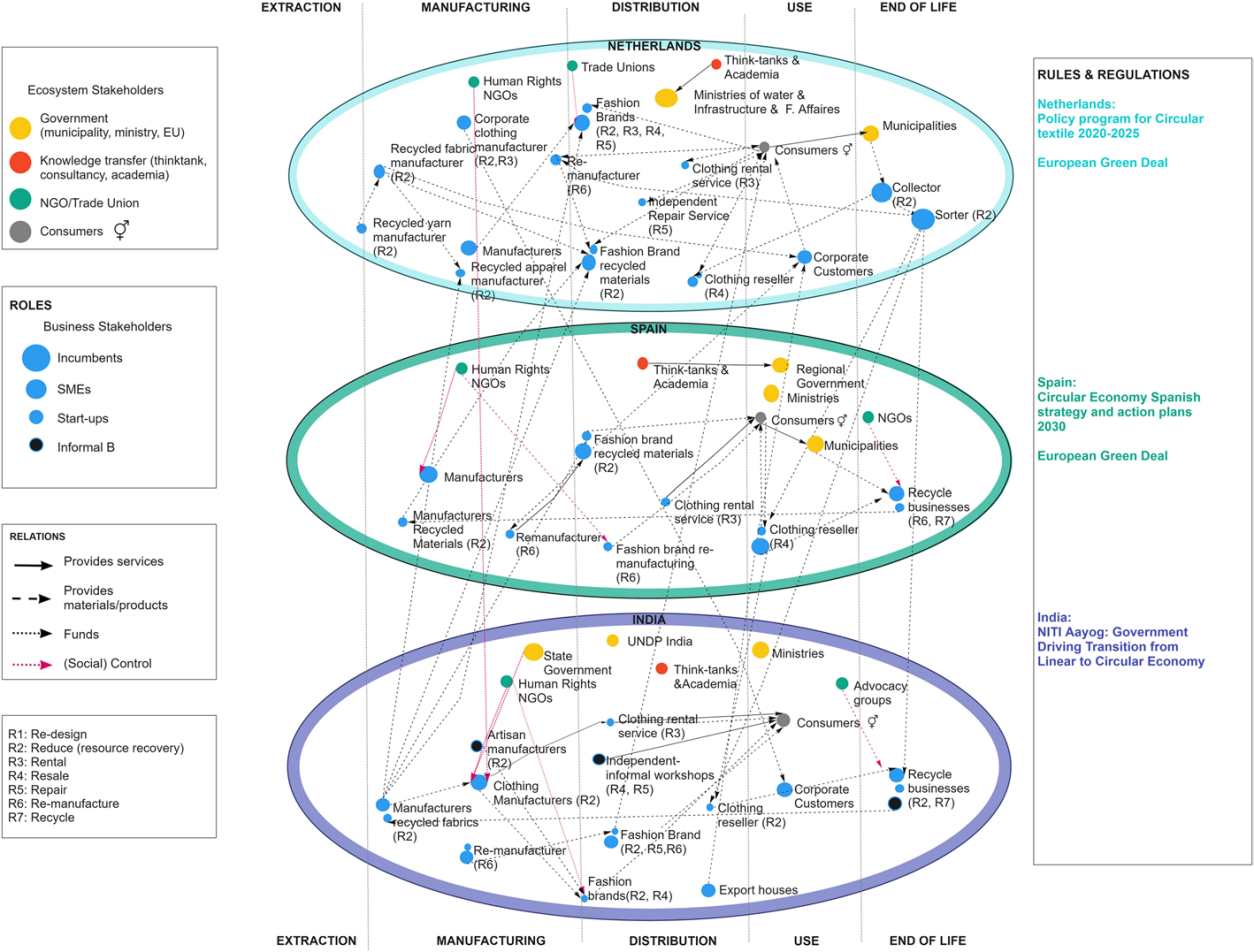
System map of the Netherlands, Spain and India, showcasing system stakeholders’ roles and their relationship

Figure 2 showcases the AVC ecosystem, including the roles and relations along different parts of the value chain. The Netherlands has several knowledge-share institutions, intersectoral associations and governmental bodies supporting circularity and employment. At the municipal level, programs such as STIP^3^ promote employment to populations distant to the job market, benefiting several social enterprises where CSs such as remanufacture and reduce are currently evolving. However, some startups believe there is a lack of financing for circular alternatives. According to (NLS09), “CSs are hard to finance in traditional banking, as rental or leasing contracts are hardly accepted as collaterals”. Additional barriers to creating decent local jobs are (i) the low increasing volume on circular demand, (ii) the higher price of circular items, (iii) the lack of manufacturing skills and (iv) the low scalability of present circular business models as put by experts (NLE02, NLE10).

In terms of CSs, resale is the most consolidated one [94]. It includes traditional brick-and-mortar stores run by NGOs, vintage and second-hand shops and B2C and C2C platforms. The Netherlands is well-positioned for resale and remanufacture because larger brands have distribution centres in Belgium or Germany. “Being close to the source of the product is critical from a timing and environmental standpoint, especially with COVID restrictions, where online sales and returns (that cannot be resold) soared and needed to be channelled some other way” (NLS07). Additionally, the in-house repair and the number of independent repair shops have grown around 20% both in the Netherlands and other global repair shops as expressed by NLC02 and NLC04. Another evolving CS is rental, characterised by the corporate (B2B) model focused on uniforms and the B2C platforms, populated by early-stage startups. They either work with a subscription model, leasing or both, paying per month or item. Finally, recycling is currently facing significant barriers to develop further because of (i) low quality of fast fashion items, (ii) low prices given the global surplus of used textiles and (iii) low consumer awareness of how to recycle clothes. According to NLC13 and NLE20, these factors impacted revenue and the number and quality of jobs available.

Different apparel circular value chain components are being developed across the European region. As put by NLE02, “companies in Italy, Spain and France are trying to set up regional cycles for recycling fibres, spinning into yarn, fabrics and garments”. Furthermore, COVID-19 has accelerated the awareness about the limitations of offshore production, which has fuelled the idea of circularity in a more regional or local sense. As NLS09 says, “Although manufacturing in Portugal or Poland is more expensive than in China or Bangladesh, I have fewer things to worry about. Because at least people here can join a union or share their stories with news reporters; here, there are checks and balances…”.

#### Spain

Spain has a generic CE policy with special provisions for the textile sector and employment and skill upgrades. These are described in the “Spanish CE strategy and action plans 2030”. Like in the Netherlands, this plan is largely influenced by the European green deal and the European strategy for circular textiles. The CE social ambition of both policy and businesses is also low and is linked mainly to local job creation. As (SPS06) said: “There is no circular economy if there are no local jobs to recirculate items”.

As seen in Fig. 2, there are NGOs, think tanks and academic institutions in the sector in terms of ecosystem development. There are also governmental programs run by the ministry of industry and economy (with circularity and innovation specific funds). However, according to SPE02, the ecosystem is not fully functional as “there is an enormous gap to articulate manufacturers with recycled textiles”. According to (SPE01) “in terms of valorising these textiles and finding an economically interesting way to sell them, there is still a lot to do”.

The most developed CSs are resale and rental with a combination of B2C and C2C platform models and traditional vintage and second-hand stores. The market is shared by NGOs and, recently, by online startups. According to a global study, resale grew 49% between 2017 and 2018 [95]. Spain is following similar trends. Repair has been revamped by innovating green tech startups promoting monthly subscription models offering repair and washing to customers, while propounding entrepreneurs franchising self-employ opportunities. Finally, recycling, in which informality is high, is regularly the subject of cases of refugees and illegal migrant exploitation [96, 97]. Several NGOs bring voice, housing and job opportunities for legal migrants, but there is a structural problem as new illegal workers arrive continuously (SPC05).

#### India

India has a CE national policy, and although the Ministry of Textiles is now promoting sustainability with initiatives such as Su-Re (sustainable revolution), textiles are not consistently prioritised in the CE national plan [98]. According to NLE10, “There is a lack of system thinking between CE ambition and policy. Even though the textile industry is very influential, it is not connected to CE policy development”, there is also not mention of a social ambition related to jobs.

In terms of ecosystem development, as seen in Fig. 2, there are also think tanks, NGOs and foreign governments supporting projects and startups related to circularity in the sector. However, according to UNEP, work needs to be more collaborative if truly transformational results are expected. Additionally, according to (INE06), “there is a lack of enabling environment to reach scale, as capital, knowledge, and networks are not entirely in place. Currently, only a few startups are paving the way”. Finally, businesses seem to agree that the road to circularity is still being built. As INS02 said, “with CSs such a remanufacturing, whatever you do is less efficient than working linearly. There are quality issues; there is no consistency in the supply”.

Based on our findings, India’s most prominent and fast-growing CSs are resale and rental, supported by a growing local market. The local online fashion rental market valued at around US$ 3–4 billion has seen exponential growth with several existing players as well as startups [99]. However, job creations and conditions do not seem to be a primary concern of startups as they are just trying to develop their circular strategy. Remanufacturing is also increasing, mainly because it is labour-intensive, giving India a competitive advantage as according to INS02, “remanufacturing in a western country, will be too expensive”. Moreover, India’s textile recycling sector which has a long tradition within Panipat region, bringing in over $62 million [100, 101], shows an increasing demand for recycled fabric and yarn, according to INC06 and INC05. Additionally, according to NLE08, “as circularity affects the creation of yarns and fabrics and as India is a significant exporter of both, there is an interesting opportunity for Indian manufacturers who have decades of experience creating recycled yarns”.

Finally, in regard to geographical and territorial aspects, it is relevant that more than 70% of interviewed businesses in all three countries operate according to the same “third-party manufacturing contracting” model in the conventional AVC. In this model, companies have only administrative staff on their payroll while a third party does their production in the same country or abroad. Incumbent businesses in the Netherlands and Spain outsource mainly to Turkey, Morocco and India, while SMEs and startups outsource to small workshops locally or regionally. At the same time, CSs as resale, rental and repair are operating locally at the city level, while incumbent businesses implementing recycling and using recycled materials are operating mainly globally, as production and repurposing of used textiles happen in Eastern Europe, Turkey and India (NLC03, NLSM01, NLC02, NLS09, NLC04). This seems to corroborate the suggestion of [102] that different CSs operate optimally at different geographic scales, but they are also replicating existing production patterns.

**Fig. 3:**
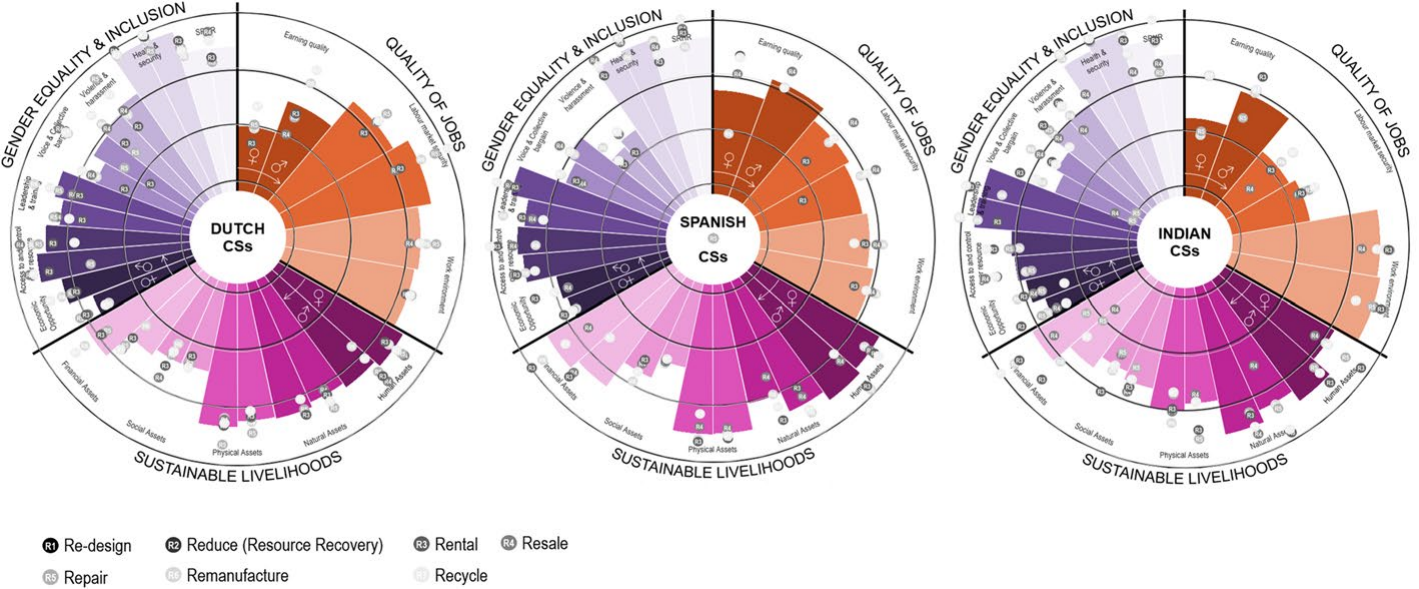
Social impacts of all circular strategies combined represented by a respective R number in the Netherlands, Spain and India

When comparing CS’s social impacts in the three countries, as seen in Fig. 3, India shows the lowest QOJ, explained by the medium–low earning quality and job security indicator of female workers, although Dutch women workers show a low earning quality especially when compared to male workers. In all three countries in the sustainable livelihood, the lowest indicators were social and financial assets in the lower spectrum of medium–high, which can be linked to the low earning and thus the low saving capacity of these workers.

GE&I is medium–high in all three countries, with health and security being the highest indicator for men and women. This score is surprising and can be explained by the fact that some of the health effects of CE result from constant exposure to chemicals over time, and are more likely noticed in the long term, while the survey health issues were considered over the last 12 months. Additionally, the fact that in the Netherlands and Spain, and until 2018 in India, every worker needs to be covered by health insurance could have also influenced workers’ response [103, 104]. Additionally, voice and collective bargaining and violence and harassment seem to be lower than other indicators. This can be partially explained by the fact that most of the businesses operating CSs are startups and SMEs with few employees who do not have a labour union. Moreover, in the Netherlands, some businesses with a CAO (collective labour agreement) for their sector were unaware of its existence, evidencing the labour union’s need to re-engage with this constituency. Additionally, the low score is also corroborated by our literature findings; these issues are hard to bring up because of shame and fear of losing their job [85, 105].

### Implementation of Circular Strategies by Types of Companies and Their Social Impact

**Fig. 4:**
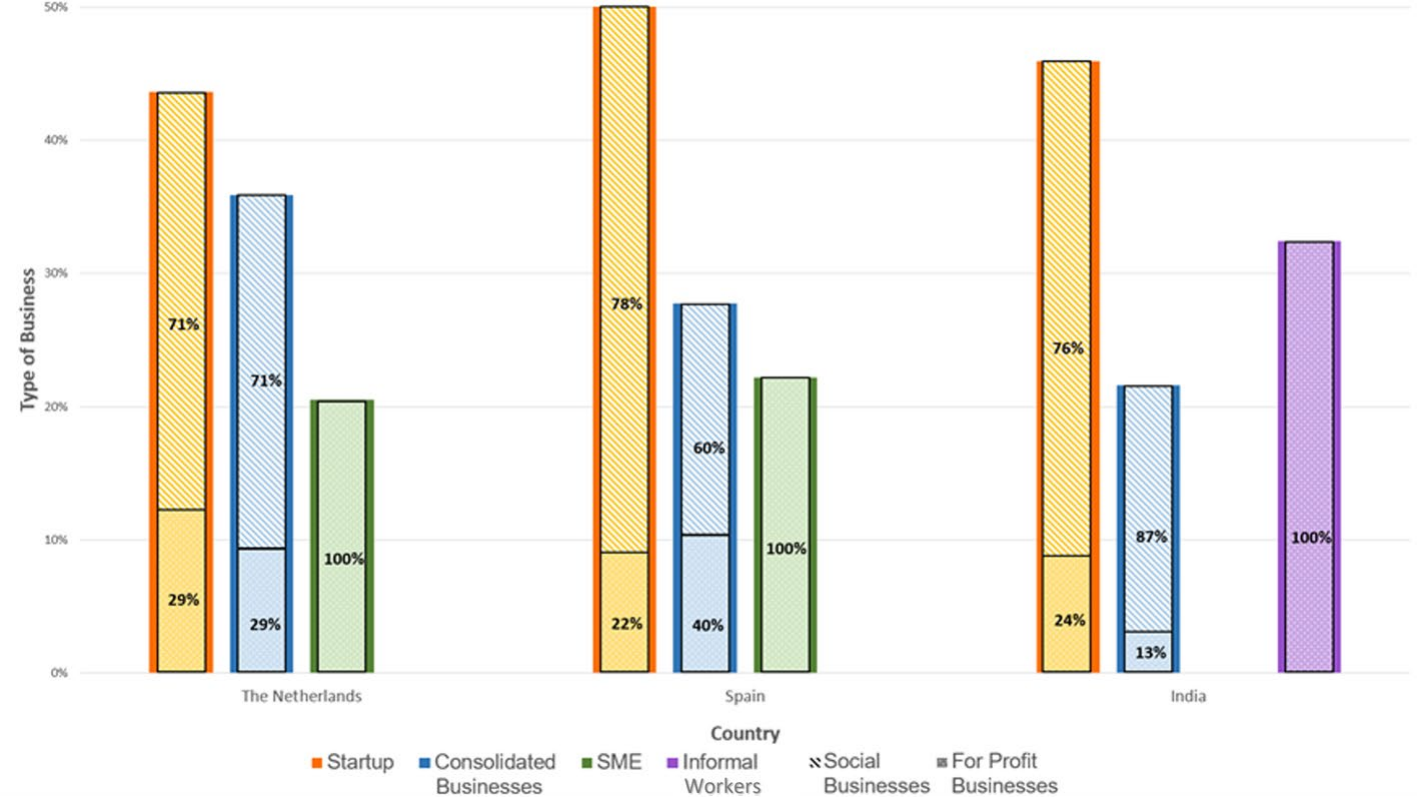
Percentage of social business and for profit business among startups, consolidated and informal workers in the three countries

Startups and SMEs are very prominent in circularity, representing 65% of our sample, as seen in Fig. 4. This corroborates literature findings that place startups as a critical actor in advancing circularity in the sector. They are also the ones advocating for local job creation.^4^ Informal workers also represent an important share of jobs in India. In our sample, they represent 32% and are concentrated in repair and resale, although literature suggests that they are also very present in the recycling phase.

It seems that when thinking of circularity, businesses primarily see the environmental angle and not so much the workers’ wellbeing.^5^

However, in all three countries, mission-driven businesses, commonly referred to as social actors (such as social businesses, NGOs and cooperatives), seem to play a critical role, as seen in Fig. 4. These actors have inclusion and integration goals in their mission and are pivotal in helping people move up the local work ladder. As put by SPC05, “social businesses have workers concern more present because they are on the core of their work”. Yet, according to NLE01, “these social training jobs can sometimes distort the system, affecting the consolidation of CSs in the sector”. These jobs are meant to train people in their employability soft skills, not hard skills like making a dress or fixing one. So, reliability in the quality of work is not always present. On the other hand, it is hard to create expertise when the demand for these local jobs is precarious. There are very few sewing ateliers in the Netherlands that can provide decent full-time employment with basic benefits, as pointed out by NLE05. It is thus pivotal to address this distortion to avoid training jobs to replace permanent jobs; ensure permanent good jobs in the sector are offered; and make sure training jobs also work on hard skills.

**Fig. 5:**
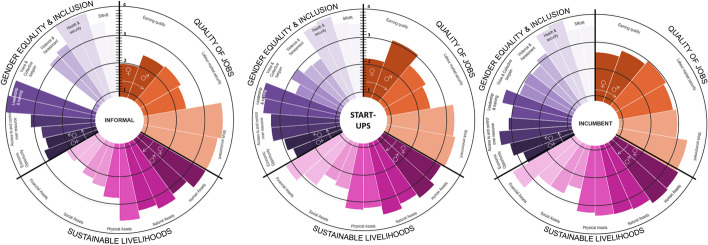
Comparison of social impacts between informal workers, incumbent and startups CS workers

Comparing different kinds of business and their social impact, Fig. 5 shows that startups’ earning quality displays contrasting differences between male and female workers, where women earn around 1/3 of their male counterparts. This pay gap is smaller in SME and incumbent businesses than in startups and informal workers. Additionally, as said by SPE02, “even though startups are born with circular DNA, most are making marginal sales volumes that create precarious jobs for entrepreneurs, which forces them to have alternative income sources”. This is true for resale and rental startups in the Netherlands, and some remanufacture startups in Spain. According to almost all indicators, incumbent businesses in all three countries provide medium–high quality jobs and reduced gender gaps. This can be explained by the fact that incumbent businesses with more than 50 employees must, by law, have a gender equality policy in Spain.

### Social Impacts of Circular Strategies for Workers

Results in this section start by describing the circular jobs and their socio-demographic characteristics and then comparing the social impact of each CS in three countries. Details for all jobs can be found in Annex 4.

#### Rental 

Typical jobs in rental include designers, logistic clerks, project managers, sales representatives and tailors (only in India). Table 3 shows the most relevant rental job characteristics per country.

**Table 3:** Most relevant rental jobs characteristics per country

Country	Manager		Logistic clerks	
	Majoritary gender	Job characteristics	Majoritary gender	Job characteristics
Netherlands	Women (60%)	Part-time (self-employed) with salaries around minimum wage	Male immigrants (90%)	Full-time
Spain	Women 60%	60% permanent	NA	NA
India	Male (90%)	60% fulltime permanent	Male (100%)	Permanent full time

In India, at B2C rental platforms, all logistical clerks and tailors are also male. This result can be explained by the fact that logistics rental jobs focused on sorting, packing and transporting heavy loads are seen as “male jobs”, as corroborated by our interviewees.^6^

**Fig. 6:**
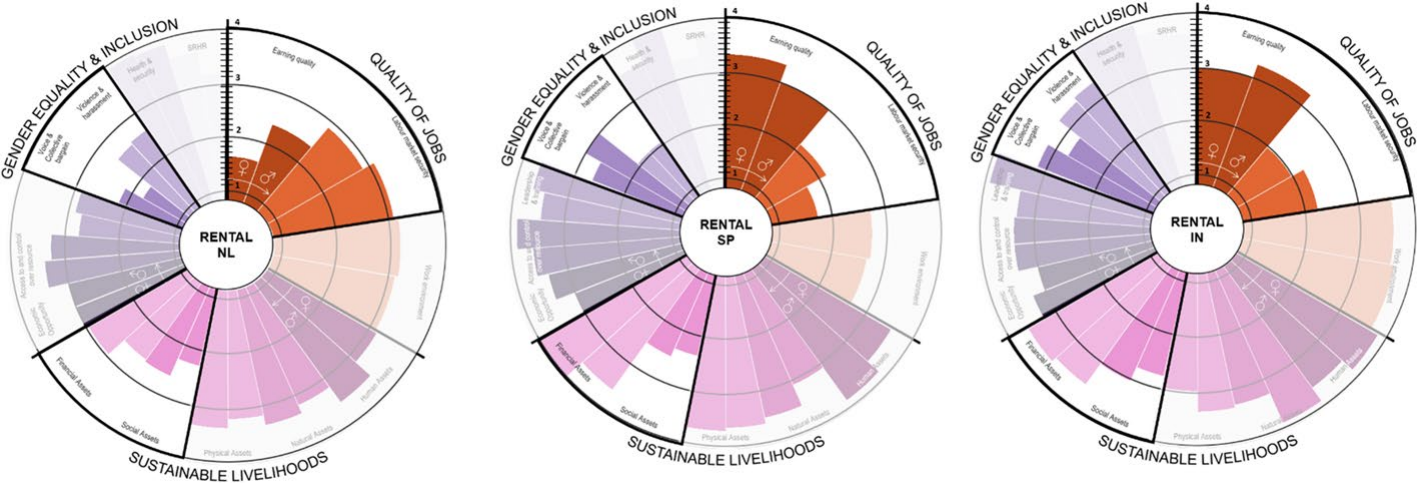
Rental’s social impacts disaggregated by gender. Relevant graph areas have been highlighted

As seen in Fig. 6, when comparing the social impacts of rental in all countries, we see contrasting realities in the *QOJ* and the *GE&I* dimensions. While in the Netherlands, *earning quality* is the lowest indicator for both female and male workers (1.7♀–2.4♂ respectively), in Spain, it is the highest for females (3.3♀), and in India for male workers (3.6♂). *SL* dimension in all three countries looks very similar, with only *financial assets* being a lot higher (3.6♀–4.0♂) for both workers in Spain and India (3.5♀–3.6♂) than in the Netherlands. In terms of *GE&I*, the most contrasting indicators are *voice and collective bargain*, which is the lowest in the Netherlands (2.2♀–1.8♂) and the highest in India (3.8♀–2.3♂). These results are surprising as one will consider the Netherlands to provide a much higher *earning quality* and better access to *financial assets*, and better *voice and collective bargain*. However, as explained in the “Implementation of Circular Strategies by Types of Companies and Their Social Impact” section, the strong presence of early startups financially consolidating their business leads to low earning quality, financial assets and collective bargain, which are lower than in Spain and India.

Additionally, rental in these two countries presents more mature startups, financially stable and more capacity to invest in their workers’ wellbeing (INS06). Finally, the high *financial assets* score in Spain can also be explained by the fact that most workers in rental are young, single female workers, who, in the case of Spain, are still living with their parents. According to INS08, “rental is the new cool job to have for young, educated women”. This statement seems to be true in the three countries, suggesting that rental currently benefits mostly white-collar workers.

#### Resale

Resale includes shop manager, shop supervisor, sales and repair assistants. Resale platforms include marketing professionals and designer stylists. In India, we also find logistic clerks and technical coordinators (Table 4).

**Table 4:** Most relevant resale jobs characteristics per country

Country	Resale platform		Resale brick and mortar	
	Majoritary gender	Job characteristics	Majoritary gender	Job characteristics
Netherlands	Women	Part-time with salaries around minimum wage	Male immigrants	Full-time
Spain	Women	Part-time	NA	NA
India	Male	30%	Male	70% informal

Most CJs in resale follow the same pattern as in the traditional retail sector. As put by NLE07, “Companies that produce abroad are occupied with the social impacts of the earlier section of their value chain, forgetting that their retail operation is also characterised with low working conditions and sometimes lack of workers' rights respect”. They are generally part-time, often short term, and pay just over minimum wage for male and female employees. Here, the work of volunteers and interns is prominent in the Netherlands and Spain. This could be explained because of the large number of NGOs and startups in a consolidation stage. In India, most jobs in resale are performed by informal workers. Resale Informal workers represent 70% of the sample, while resale startup platforms represent the other 30% of which half are male workers.

**Fig. 7:**
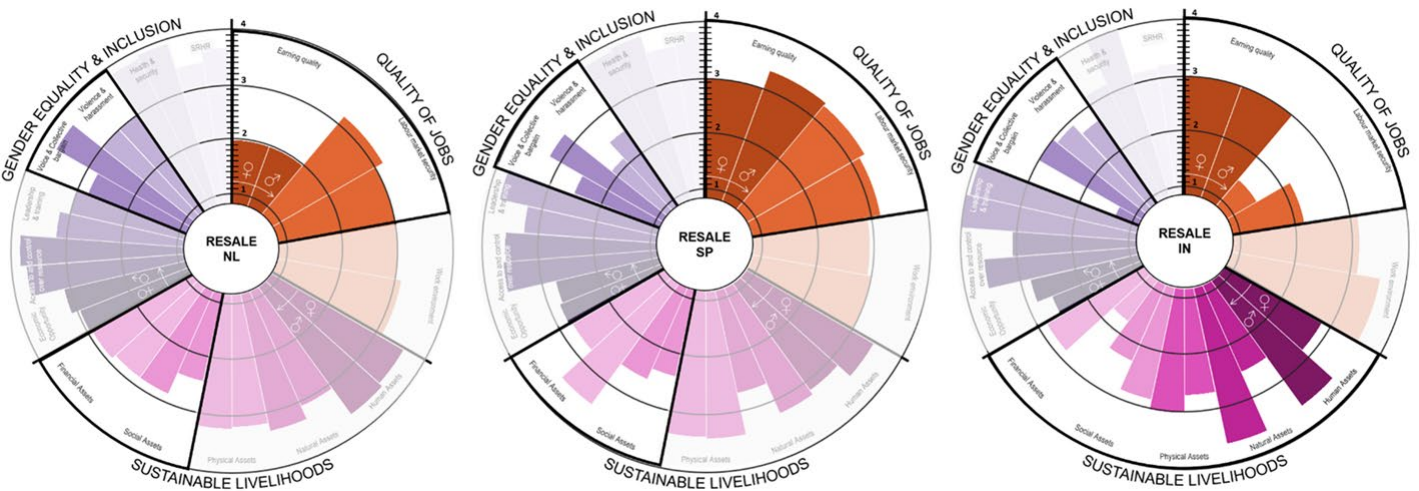
Resale social impacts disaggregated by gender

Social impacts show contrast among different countries. In the Netherlands, *earning quality* is *medium–low* (2) for both female and male workers, while in Spain and India is *medium–high* (3.0♀–3.3♂). In both countries job *security* is relatively *low* for India (1.50♀–2.2♂) compared with both Spain (3.3♀–3.2♂) and the Netherlands (3.2♀–3.0♂). Regarding sustainable livelihoods, while resale in the Netherlands and Spain scored similarly for all workers in most indicators, the contrast between male and female workers is more pronounced in India. As seen in Fig. 7, women score lower in *human*, *natural*, *physical* and *financial assets*. This can be explained by the lower presence of collective agreements that guarantee higher-than-average working conditions regarding vacation, health and pension schemes, as corroborated by our interviewees (INE03).

Regarding GE&I in India, *voice* and *bargain* are significantly lower for Indian female workers due to the high informality level in the sector and early startups.

#### Repair

Repair comprises tailors, designers, sales assistants and, specific to India, cobblers. Companies, where repair is performed in-house, do not create additional jobs. Detailed characteristics of repair job can be seen in Table 5.

**Table 5:** Most relevant repair jobs characteristics per country

Country	In-house sales repair jobs		Independent repair shops	
	Majoritary gender	Job characteristics	Majoritary gender	Job characteristics
Netherlands	Women (66%)	Part-time with salaries around minimum wage	Male immigrants (67%)	Full-time self-employed
Spain			Women	
India	Male (72%)	Full time (88% overtime)	Males (72%)	Self-employed or informal

**Fig. 8:**
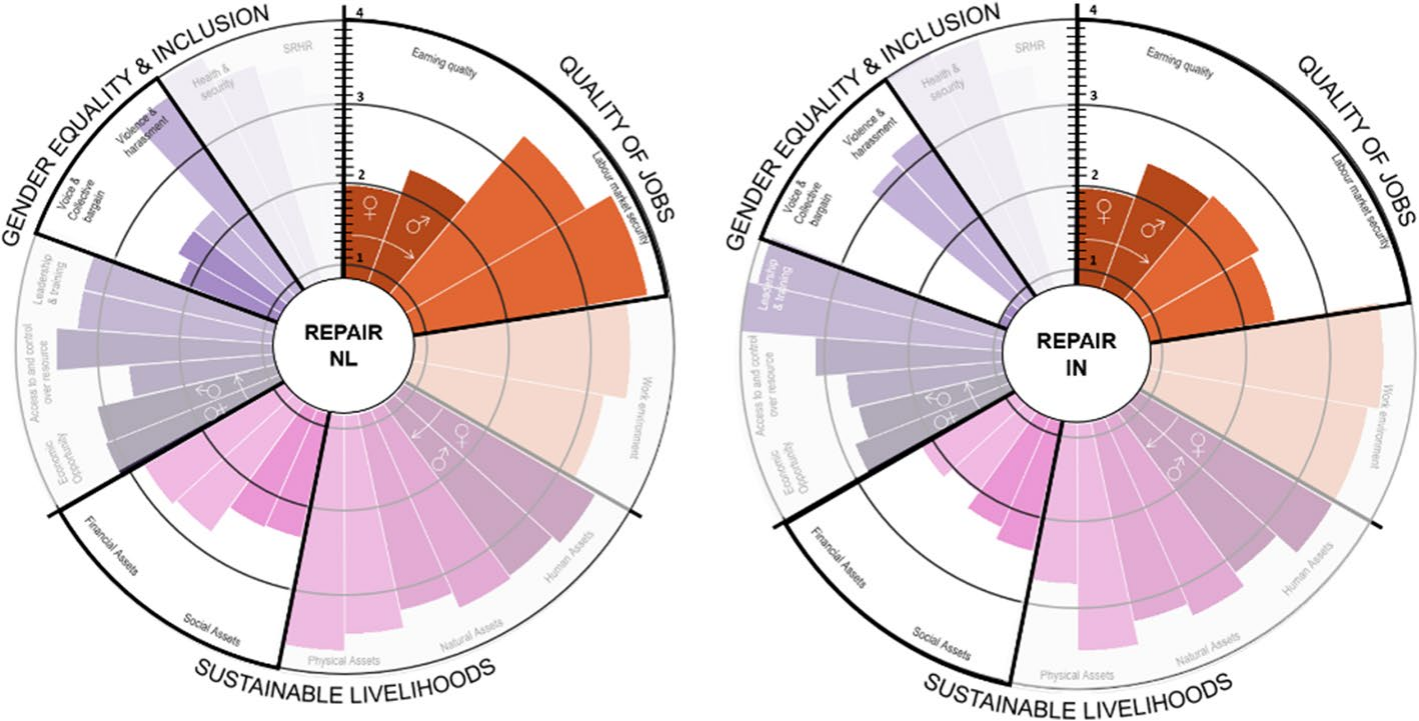
Repair’s social impacts disaggregated by country and gender

When comparing repair in the Netherlands and India, as shown in Fig. 8, we see contrasting realities mainly in *earning quality*, *labour security*, *financial assets*, *voice* and *bargain* indicators. In both countries, *earning quality* is closer to the minimum wage (medium–low) and lower for female workers. Although in the Netherlands, the minimum wage is very close to the living wage, it is still considered in the lower pay range of the sector. According to experts in India, female workers earn below the minimum wage, which is insufficient to cover basic needs (INE03*). Labour security* is significantly lower in India than in the Netherlands, while *voice and collective bargain* are the lowest indicators (1.0). Both results can be explained due to the informality nature of these jobs in India. However, in terms of *work environment*, workers in both countries perceived their working environment as favourable (3.4♀ and 3.5♂).

Regarding *violence* and harassment, in the Netherlands, male workers consider that their employers have appropriate policies, while women workers do not (2.3♀ and 3.5♂). In India, this indicator shows score of (3.1♀–3.3♂). These results corroborate our literature findings that *violence* and *harassment* is still hard to speak about in the AVC, reflecting broader societal issues in Europe and India [106, 107].

#### Remanufacture

Remanufacture includes tailors, designers and sewing machine operators. In Spain, it also includes buyers, managers and sales assistants, while in India, the sector also employs clippers and logistic clerks (Table 6).

**Table 6:** Most relevant remanufacture jobs characteristics per country

Country	Machine operator		Tailors	
	Majoritary gender	Job characteristics	Majoritary gender	Job characteristics
Netherlands	Women (75%)	Part-time with salaries around minimum wage	Male immigrants (90%)	Full-time self-employed
Spain	Women (78%)		Women (78%)	Part-time and permanent
India	Women (64%)	Full time (88% overtime)	Women (50%)	Informal workers

**Fig. 9:**
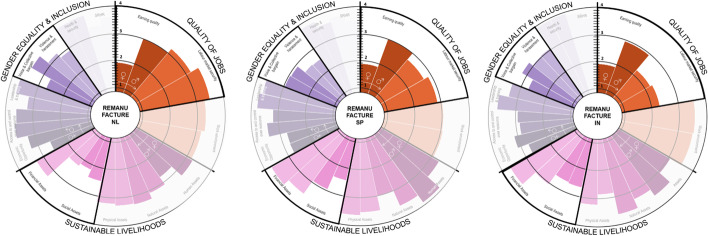
Remanufacture’s social impacts disaggregated by country and gender

Regarding QOJ, we found pronounced pay gaps between male and female workers in the three countries. As seen in Fig. 9, male *earning quality* is 50% higher than females. Also in the Netherlands, 37% of remanufacture workers are divorced women, which adds an extra burden for their family obligations, as they mainly work part-time and make less money. In all three countries, men have the highest paying positions. This is linked to a higher score in *financial assets* as well. This strongly relates to gendered job segmentation as put by INS03, “In India, males are the skilled tailors, the pattern cutters while women are often employed in low skilled line-work”. In terms of GE&I, while *economic opportunity*, *voice and bargain* and *violence and harassment* scored medium–high, they scored slightly lower for women workers in all three countries.

#### Recycle

The most relevant jobs in recycling are sorter, logistic manager, logistic clerk and buyer. Table 7 shows most relevant jobs and characteristics.

**Table 7:** Most relevant recycle jobs characteristics per country

Country	Sorters (clipper in India)		Logistic clerk	
	Majoritary gender	Job characteristics	Majoritary gender	Job characteristics
Netherlands	Women 67% (60% immigrant)	56% permanent, 66% part-time with salaries around minimum wage	Women 67% (45% immigrant)	56% permanent, 66% part-time with salaries around minimum wage
Spain	N/A	NA	NA	NA
India	Male (100%)	Permanent, fulltime	Male 100%	Permanent, fulltime

**Fig. 10:**
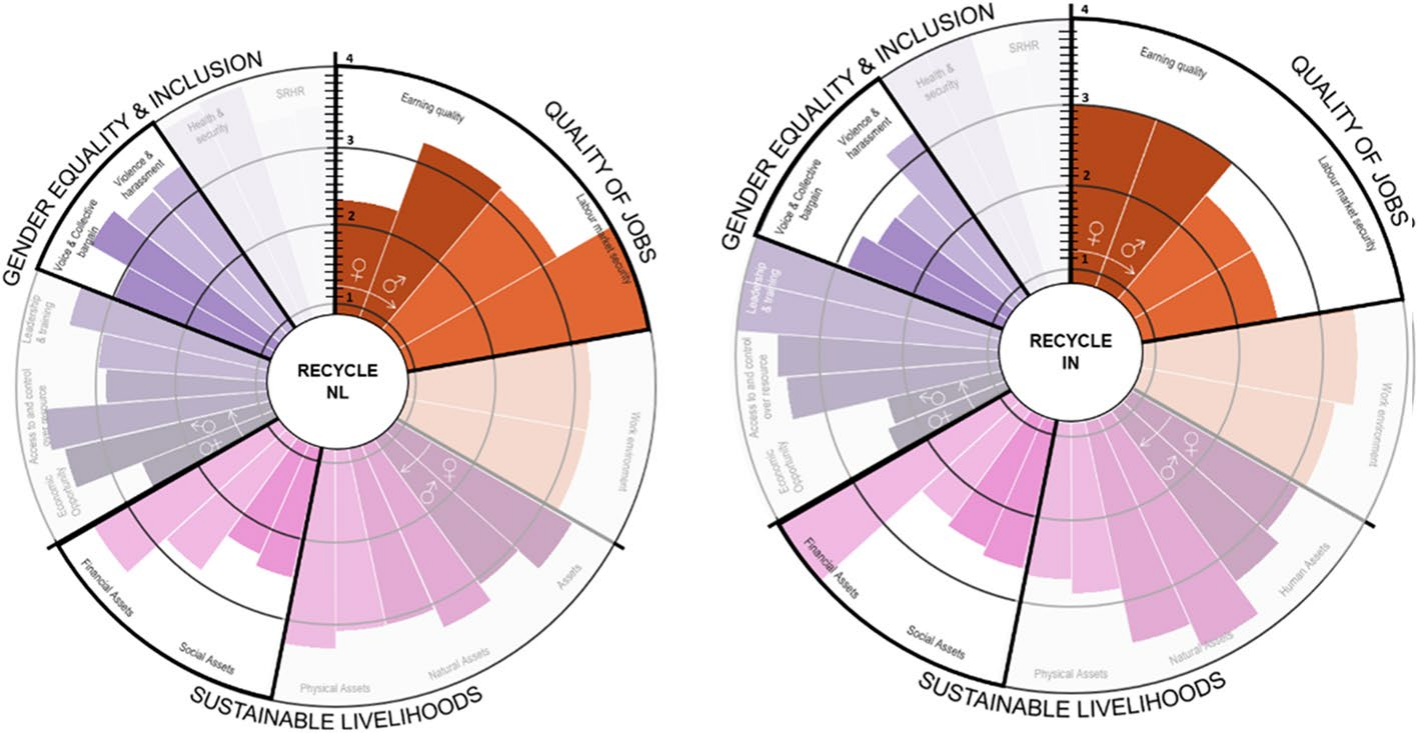
Recycle’s social impacts disaggregated by country and gender

In India, recycling is characterised by a high number of informal workers. However, due to COVID-19, most respondents come from formalised employment. The *earning quality* in the Netherlands is significantly lower for women than for men (see Fig. 10), the highest-*earning quality* for women in India as most workers are formalised. In terms of *sustainable livelihood*, women have significantly lower *financial assets*, which is explained by a higher amount of family debt and a small capacity to save, as confirmed by workers’ surveys. Even though the sorting job was mentioned as a pivotal job for the sector (NLC09, NLC13, NLC10), sorters are paid far less than the clerical logistics position, demonstrating the lack of value the recycler position gets. Sorters earn around the minimum salary. However, according to management, sometimes sorters get a bonus for sorting of high quality, but as put by NLC13, NLC10 “if your bonus relies on the quality of sorted products, and you have bad bags because of dirt or quality of items, your final pay suffers in the end”. The *sustainable livelihood* dimension is considered *medium–high.* However, the financial assets indicator shows a considerable variation between male and female workers, where *financial assets* show a lack of saving and debt management in the household of female workers.

In contrast, access to *financial *assets is the highest indicator for males in India and the second-highest for males in the Netherlands, evidencing of higher *economic capacity.*

#### Intersectionality Analysis

In terms of intersectionality, gender, migration status, language/race and class-caste intersect in CSs, marginalising more vulnerable populations. The most vulnerable workers in terms of QOJ, SL and GE&I are informal workers in repair and remanufacture, as evidenced by the low earning quality and low SL. However, in our sample, we did not have informal enterprises as presented in [108]. In the informal category, we only had informal repair and resale independent workers. For instance in India, while dry cleaners and tailors (often males) have predefined rates, the cobbler (informal worker and often woman) is paid per assignment and must negotiate with clients each time. More so, informal women refugees in resale who often come from a caste, ethnic or religious minorities and do not speak the local language are not even covered by basic national Indian insurance, as corroborated by workers surveys, making them an even more vulnerable worker. Informal women workers in resale and repair have greater access and control of economic resources, as they were making financial decisions (autonomy) of their household. They also have a high perception of working conditions as they can decide their working schedules. However, their access to financial assets is very restricted (as their income is very low and they have very limited capacity to save). So even though their agency and autonomy are higher, due to their low income and low access to labour security, they are the most vulnerable of workers when comparing the different circular strategies in India under the SIAF-CE lens.

According to INE02 and INE04, there is a need to ensure that circularity benefits both formal and informal workers, especially the unorganised textile waste picker, the most vulnerable workers of all. They have no voice and no negotiation power, so they are exploited by more prominent dealers.^7^ To reduce vulnerability in informal workers, a transition to formalisation should be accompanied by a process in which they are organised (in an community association or a so call informal community enterprise) to have a collective voice that represents them [108].

In formalised settings, immigrant workers, especially women, are also prone to salary discrimination for the same job and same years of experience. This was noticeable, especially in a few startups in Spain, where salary scales are not standard, and salaries are negotiated when being hired. In the Netherlands and Spain, “newcomer” workers are served by a well-established network of re-integration social enterprises, which play a pivotal role in helping these workers to enter the job market. However, as SPC04 said, “the biggest challenge of social enterprises is to continue helping migrants when they find a job because even though they are integrated into the society, they are also excluded from it. As they are relegated to the types of jobs that nobody else wants to do”. This situation can create a social trap for these workers. Ideally, circularity will help people gain new skills from which higher salaries and opportunities are present too.

Additionally, there is an apparent asymmetry in how we value professions, which is influenced by gendered cultural norms [24, 27, 109]. This is evident in the AVC, where “work” value is based not on quality but on the title of who performs it. As SPS07 said, even though men and women tailors do the same job and have the same skillset and knowledge, there are different levels of perceptions of both types of workers. For instance, in the three countries investigated, a tailor is generally male. The position is perceived as a master craftmanship which is better paid. Female tailors are in Spain considered “dressmakers”; they have good craft skills and are paid less than male tailors. Likewise, repair assistants and cobblers in India are mainly women or people from lower casts working informally and making less money.

Moreover, the same tailor position has a significant earning contrast based on who (which type of company) pays for it. For instance, In India, the luxury rental sector, where tailors are always male, is where the highest salaries are (even though these tailors are not formally educated). In contrast, women workers in remanufacturing holding the same skillset and position are the lowest paid. Finally, in the Netherlands, as put by NLE01, “jobs like repair and (re)manufacture are not very highly viewed either in a social sense or financial sense. People want to be designers”. At the same time, as students today are not taught to put a dress together, there is a current lack of tailor’s skills. Interestingly, our results also point out that tailor jobs are currently the most relevant for circularity in all three countries as they are required for repair, remanufacture, resale and even rental. This situation evidences a deep value gap for the profession, associated with traditional gendered structures of the traditional AVC, which are also being perpetuated with CSs in the sector.

## Discussion

### Social Impact of Circular Strategies

Today, rental and resale are the most thriving strategies in the three countries, which depend on a growing local urban middle class with increasing purchasing power and consumer awareness. They also showed the highest number medium–high scores demonstrating a better social impact than other strategies. Additionally, rental seems to generate more jobs because it incorporates repair, maintenance and resale, representing an opportunity to explore local and regional CE approaches. Nevertheless, according to our findings, rental, repair and resale share the same characteristics typical of the retail industry, e.g. short contracts, minimum wage payments and sometimes part-time jobs (even for educated workers). Also, as these circular strategies depend on a high transport logistics online-platform model, there is a growing concern that low social protection might arise, as it has also been observed in the so-called GIG economy [110–112]. Additionally, social issues such as gender gap disparity, poor working conditions and low collective voice and bargain can also be observed in remanufacturing and recycling CSs. As in the linear AVC, they also show a growing presence of undocumented refugees or migrant workers.

Tailoring jobs are currently the most relevant for circularity as they are required for repair, remanufacture, resale and rental. According to our interviews, immigrants and refugees in Spain and the Netherlands are bringing back these skills and knowledge to circularity, as the offshoring production trend in the industry since the nineties made these skills in the local industry obsolete. However, they seem to have lower QOJ and lower family wellbeing while they are often stigmatised as low-value positions, as pointed out by NLE07. This situation reflects the need to increase the professionalisation of tailors through technical vocational schools. These schools should include specialisations and re-skilling such as dressmakers, menders, remanufacturers and de-manufacturers. In addition, a revalorisation campaign should be created to de-stigmatise and de-genderise the profession. Such campaign will promote dignity and value in professions like repair and remanufacture. It should have an internal (within companies) and external focus (with potential job candidates and wider society as a whole). Companies’ internal campaigns should aim to give these jobs more desirable attributes such as better pay, better contracts agreement with more flexible schedules and learning opportunities to grow.

The external revalorisation campaign should be a joint effort from the sector and employment government agencies showcasing the added value of these professions in vocational training and fashion schools, in career job fairs and on social media. A curated team for gender and inclusion should also make sure that the campaign speaks to all types of citizens that are diverse and inclusive in the use of its messages and visuals*.*

### Companies Applying Circular Strategies

Companies applying CSs reproduce the same third-party contracting model as the traditional AVC. Although outsourcing allows businesses to grow their brand by leaving the manufacturing to others, third-party contractors can only provide basic income and temporary contracts to their workers as they depend on brands orders. This model keeps workers powerless, poor, insecure and vulnerable to all market changes. This was evidenced during COVID-19, when cancellation of orders, lack of payment and illegal firing of employees occurred [4].

Also, although circular incumbents and startups often outsource activities from the same supplier of linear AVC, allowing them to build a long(er)-term relation, collaborate and substantially improve the suppliers’ working conditions, these efforts concern only a small fraction of the industry. They lack a systemic approach where benefits ripple down to the most vulnerable workers of the sector. Finally, our results show that *labour security* is lower when both informality and startups are present, which is the situation for current CSs in remanufacturing, repair, resale and recycling. This point is critical because if these CS increase as circularity become more established, workers’ job security and access to unemployment benefits will be affected. In this case, impact capital seems to play a role in stimulating positive social impacts as the Indian startups supported by “sustainable ventures capital firms” provided to their employee’s permanent contracts and benefits such as transportation, education allowances, pension and savings through schemes as the provident fund and the Employment State Insurance (ESI).

### CE Policy 

Circular economy policies adopted in Spain and the Netherlands have a low social ambition which currently only mentions job creation potential. Additionally, these CE policies seem to be disconnected from producing countries such as India. On one side, European CE policy advocates for a reduced demand for primary sources and new products, supported by SMEs and startup European companies with a strong emphasis on “creating local jobs”. On the other side, neither policymakers nor businesses in India seem to be aware of the potential trade-offs circularity might bring to the Indian businesses and workers, as they are more concerned with addressing production issues. A more global systemic level perspective is needed, connecting stakeholders at local and global realities. This perspective is pivotal for closing different performance loops and ensuring a fair transition.

### Basis for Policymaker and Industry Recommendations

For circularity to be genuinely transformational and contribute to sustainable development, it must address several issues:

1. It should have a more substantial social paragraph in its definition, elaborating on the quantity and quality of jobs created, the direct and indirect impact for communities in which those CSs are implemented, and how CS can affect gender and inclusion imbalances. The inclusion of a social perspective should be an effort carried out by policymakers and businesses.
2. It should acknowledge the structural imbalances of the AVC, and therefore re-imagine an inclusive circular fashion system aimed at equal opportunities for most disadvantaged workers. This could be accomplished by (i) mandatory human rights due diligence (including the incorporation of workers’ committees in all negotiation), (ii) privileging more extended contracts (and incentivise them) and (iii) requiring companies to extend transparency efforts up to the informal worker by gathering specific gender statistics supported by the NGO working with these communities and promoting the development of plans to incorporate these workers in a more democratic and formal way. As pointed out by several interviewees, businesses should work more integrally with vocational training centres to ensure that workers have the right set of hard skills.
3. Policymakers should ensure the ratification and implementation of international labour standards and develop incentive programs for startups to adopt early-on social and environmental assessing mechanisms. Companies should be required to report on both environmental and social performances of circularity. Additionally, NGOs and trade unions should provide subsidised awareness-raising and training in every company (disregarding the size or type) with a particular emphasis on migrant workers. This will ensure all workers understand their rights, the internal company procedure and the external normative provision at their disposal regarding violence and harassment, voice and collective bargaining.
4. Coordinated policy promoting inclusive CE should encompass different governmental bodies at various levels that work within the countries and across the different stages of the AVC. For instance, the labour and finance ministry and the ministry of international affairs should work together with the ministry in charge of mobilising efforts to improve circularity. Ideally, this should be done at municipal, national and European level, coordinating international bodies of the apparel value chains.

We acknowledge that these bases for recommendations will not radically improve the poor working conditions along the AVC. We hope that the suggested basis for recommendations supports the endeavours of all stakeholders to (re)create a sustainable and equitable value chain.

### Limitations

Research limitations are related to either survey size and population or scope.

It is important to note that the social impacts covered in this research are not exclusively attributable to CSs as they are also the result of current national regulations, policies and cultural/sectoral norms. However, the labour intensity of CSs in the AVC can further exacerbate current poor working conditions if the uptake of circularity continues to grow in the sector. Taking a worker’s perspective meant that survey responses were based on workers’ perceptions which could also carry some bias. Additionally, companies provided the logistics and space to conduct surveys, possibly influencing workers’ responses to more positive outcomes.

COVID-19 restrictions affected the number and type of workers interviewed. Not all surveys could be conducted in person, and some workers neither had regular access to the internet nor felt comfortable answering personal questions to a screen. Also, not all platform-based rental businesses participate in interviews or surveys, which might play a role in the low earning quality reported in the Netherlands. In Spain, repair surveys were insufficient, and it proved impossible to reach informal textile waste pickers in India.

The limited sample size (210 workers surveyed) provided a partial understanding of the challenges and perspectives for workers in the industry.

### Future Research 

This research provided evidence of the current social impacts of CSs in the AVC, highlighting the risks and trade-offs. Future research should look at how CSs can increase the quality of jobs, workers’ sustainable livelihood and gender equality in future scenarios where circularity uptake is growing at global scale. This paper aimed to set some ground basis for recommendations. However, more in-depth recommendations both for policymakers and businesses to improve the deployment of more inclusive circular practices in the sector should be the focus of future research.

Additionally, in our sample, businesses carrying a social mission seemed to accommodate better distinct workers’ needs. Future research should analyse this further by comparing how different corporate structures and management styles affect the social impacts of circularity.

## Conclusions

This study addresses the lack of knowledge about the social impact of the different CSs implemented in the AVC. Using a mixed-method approach and a novel social impact assessment framework called SIAF-CE⚥, it addresses the social impact of CSs in the Netherlands, Spain and India from the perspective of workers and different company types.

From a country perspective, the current social ambition of CE is low in all three countries analysed, and it does not provide any attention for the quality aspect of jobs created. Current circular jobs seem to follow the linear AVC in terms of behaviours and practices and reproduce the same social imbalances and inequalities we see throughout the AVC today. Earning quality is between minimum and living wages in all three countries, with India having the lowest QOJ. Sustainable livelihood is sufficient in all three countries for male and female workers except for social and financial assets, which are lower than the other indicators. While most gender equality indicators score medium–high, these are generally lower in India, especially for *violence and harassment and voice and collective bargain*. Additionally, this low participation in labour unions and general survey participants’ disengagement around their collective working conditions, coupled with the third-party contracting model, is worrisome, as it keeps current asymmetrical power relationships among workers and companies along the supply chain. This could be exacerbated if circularity increases without fully addressing this issue.

CSs seem to be at a very early adoption stage for incumbents and startups, with the deployment of CS happening organically. In terms of social impacts, startups show contrasting earnings between men and women, with women earning significantly less. In comparison, incumbent businesses provide medium–high quality jobs and reduce gender gaps. Since startups are driving circular efforts and creating local jobs, it is pivotal to develop incentives to help them strengthen their social impact ambition and social impact in their circular activities.

From the workers’ perspective, current CSs are replicating the feminisation trajectory of the linear AVC, with women being overrepresented in the jobs with the lowest salary and poorest working conditions from manufacturing to the end-of-life segments. Moreover, decent working conditions for informal workers and migrant women workers are still far off with circular practices employed today in the industry. Overall, women experienced lower social impact benefits than their male counterparts in most indicators across the three countries.

Policymakers and businesses of the AVC need to strengthen their CE social impact ambition and harmonise policy and strategies along different geographies to minimise trade-offs and safeguard a just circular transition for all.

Finally, this study demonstrates how the SIAF-CE⚥ provides a solution to the lack of gender-disaggregated data in the AVC. It also provides the basis to assess the social impact of the CE, which allows to understand and minimise trade-offs of jobs in different stages and geographical locations of the AVC. Moreover, by providing evidence of the social impact of current CSs, it provides basis for industry and policy recommendations.

## Supplementary Information

Below is the link to the electronic supplementary material.

Supplementary file1: Annex 1. The social impacts of the linear apparel value chain (DOCX 41.1 KB)

Supplementary file2: Annex 2. Methodology Appendix (DOCX 14.4 KB)

Supplementary file3: Annex 3. Survey SIAF-CE⚥ (DOCX 20.4 KB)

Supplementary file4: Annex 4. Circular Jobs Inventory and gender baseline (DOCX 156 KB)

## Data Availability

The data (except sensitive information) is available upon request to the corresponding author on reasonable request.

## Footnotes

1 Around 6 years of existence according to the OECD definition.

2 Intersectionality analyses how gender inequities manifest within a particular social context, how they intersect with other social stratifiers such as race, class, migration status and the compound effect this intersection has on the quality of their jobs, their livelihood and inclusion of different workers. Intersectionality does not have a prescriptive process but suggests approaches adapted to the context [138].

3 Acronym in Dutch for jobs with a social pathway in perspective.

4 NLS10: “It does not make sense to use local post-consumer material if it travels four times around the world to be processed, transformed, and manufactured before we have it back into our clothes”.

5 NLE01: “There is just not a huge awareness about people working on circular business models, nor in how they should begin to approach the topic and the success criteria”.

6 (INS04) said: "in our warehouse, most employees are men because of tough schedules and driving distances." In The Netherlands, (NLC01) said: "even though technically both men and women could do this job we have never gotten a female applicant".

7 As INE05 said, “they suffer all sort of social stigmas such as interfamily violence and drug addiction. They are by far the outcast of the system, and if we do not do something to incorporate them fundamentally, there will be the outcast of the circular economy too”.
