# Supplementary material for: The Social Impacts of Circular Strategies in the Apparel Value Chain; a Comparative Study Between Three Countries
Source: Circ Econ Sustain. 2022 Sep 6:1–34. Online ahead of print. doi: 10.1007/s43615-022-00203-8 (PMC9446664; doi:10.1007/s43615-022-00203-8)
Supplement: Supplementary file 1 — Supplementary file1: Annex 1. The social impacts of the linear apparel value chain (DOCX 41.1 KB) [file 43615_2022_203_MOESM1_ESM.pdf]

Annex 1 The social impacts of the linear apparel value chain

Social Impact		Traditional linear AVC		
		Manufacturing	Retail	End-of-Life
Number of Jobs	Europe	Textiles and apparel account for 6% of total employment in manufacturing [106], while textile apparel create more than 1,7 million jobs [107].		N/A
	India	the AVC provides more than 45 million direct jobs and 55 million indirect jobs [108]		
Earning quality	Europe	Payment around minimum wage [109]	Retail workers USD29,000 on average [65]<br>Low average wage €1,112 monthly in 2015 [66]	Recyclers $29,000 annual average . Payment is low [65]
	India	Average wage rates are 50-60 % lower than that in developed countries [110]<br>Living wage is paid by a minority of companies, most companies paid minimum wage [68]<br>Home workers are paid by piece	Retail workers earn average $1,439<br>while sales executives earn an average of INR 18,197 per month[111]	Recyclers earn $2,064 a month while ragpicker earns about Rs 40-50 a day. [112]
Type of contracts	Europe	Part-time workers are the 14.3% of total textile manufacture workforce [113] Short-term contract with no guarantees and security especially in lower income European Countries [114]	High presence of casual work, short term, part timework, zero hours work (32% in Europe) [66]	Contracts of limited duration [67]
	India	Workers are predominantly (internal) migrants from the poorest households in deprived rural and urban areas. [115]	Most contracts are fix contracts. Shop assistants are often paid less than the minimum wage and forced to work seven days a week. [116]	High level of informality and non-standard forms of employment [117]
Working Conditions	Europe	40 hours is a fulltime job<br>Minimum wage<br>In least developed european countries Even after 20 years working in the factory some women don't get overtime bonus paid according to the law.<br>afraid to speak about working conditions for risk of losing their job. [118, 119]	37,5-40 hours is a fulltime job<br>Atypical working hours<br>Not a lot of paid training by employeers<br>Few possibilities for career work<br>[66]	37,5-40 hours is a fulltime job<br>workers have to deal with all kinds of materials, and in the treatment process(toxic)substances.<br>High physical demands<br>Low social dialogue. Employee representaion is difficult.<br>unequal working conditions for new entrants and fragmentation of workforce [67]
	India	48 hours is fulltime. High level of overtime. Low voice and collective bargain [120]<br>Low compliance with basic safety procedures. badly ventilated work areas, run-down factory building conditions [121]<br>high Exposure to toxic chemical dyes and sandblasting which are harmful for human health [122]<br>high level of informality, especially homeworkers.	Not being able to sit down.<br>constant surveillance by managers and facing restrictions to use the bathroom. [116]	engaged in labour intensive work, associated with lower rates of utilisation of technology, and with fewer provisions of financial security. [117]
Gender Equality	Europe	EU: 69.4 % of workers are female In several european countries gender pay gap in the industry range from 40.4% to 7.3%. [113]	70.8% women [123], part-time female-dominated retail sector [66]	More women working in the sector than men. Women are working predominantly in sorting post. [124]
	India	80% of workers are women. They earn 57 rupees for every 100 earned by men. They have limited legal protection, weak trade union representation and 38% of them have to deal with sexual harassment in the workplace.[125]	Retail industry has opened employment opportunities for women. however, women workers typically in sales job and marketing and they are poorly paid. [126]	Waste picking is often a family enterprise. It offers flexible working hours (especially important for women) and a high level of adaptability. [127]
Inclusion	Europe	Migrant workers constitute a significant part of the workforce, but they are also the most vulnerable to exploitation, discrimination, and lower salaries [128]	64% of workers in retail in europe are women<br>High presence of younger workers<br>It employs more low qualified workers [66]	High presence of immigrants as well as undocumented informal workers paid in cash special cases in murcia.<br>[92, 124]
	India	high presence of lower caste, (Dalit) undocumented migrants. An estimated 100,000 young children and teenage girls are victims of 'bonded labour' or 'modern slavery'. [129]	The apparel business is still largely unorganized, with formal retail accounting for just 35 percent of sales in 2016. [130]	Work done primarily by disadvantage groups Refugees, undocumented migrants lower cast workers [127]
