# Supplementary material for: The Social Impacts of Circular Strategies in the Apparel Value Chain; a Comparative Study Between Three Countries
Source: Circ Econ Sustain. 2022 Sep 6:1–34. Online ahead of print. doi: 10.1007/s43615-022-00203-8 (PMC9446664; doi:10.1007/s43615-022-00203-8)
Supplement: Supplementary file 2 — Supplementary file2: Annex 2. Methodology Appendix (DOCX 14.4 KB) [file 43615_2022_203_MOESM2_ESM.docx]

**Annex 2 Methodology Appendix**

**About semi-structure interviews:**

The interview was divided into three sections. Section 1 referred to generic aspects of the company in terms of circularity plan, what types of CS, their processes, and their general operating structure. The second section referred to the social impact, how they measure it, under what criteria and why they were there not assessed if they were not assessed. The third section asks about contracts, the hiring process, gender equality, inclusion policies and working conditions and special training and leadership programs. The interview guide is included in annex 2

The thematic analysis originated from the SIAF-CE dimensions. They guided coding for semi-structured interviews. They were also organised by subthemes (given by the indicators of each dimension). Other themes included power dynamics and social-cultural context. When new themes were not considered in pre-selected themes, they were created and added. A clustering by country and similar themes per dimension was the last analysis stage.

**About Intersectionality analysis method:**

*As intersectionality does not prescribe an analysis method per-se, (Weldon 2006;* McCall, 2005) *we followed a three-step* method. First, we use the gender intersectionality matrix design by the (WHO; 2020) to identify the relevant social(vulnerability) categories for our research which in this case were (Gender, education, ethnicity-migration status). As Hunting (2014) points out, it is important to reconceptualise the meaning and the consequence of the categories that are used during the research. Second, we plotted the four gender relation domains (related to access, agency, and empowerment) i) *access to resources, ii) distribution of labour, iii) norms & values, and iv) decision-making power, against the social impact domains we are interested in investigating (QOJ, SL and GEI). Third, using gender as the first and the guiding social stratifiers, we use the “other question” method. The other question method (Matsuda, 1991) suggests asking the “other question” first to a group of x individuals with one category, and then adding one other categories and ask the question again, until all the intersection of categories and ang gender domains established are considered. So first, we asked how in the category of gender; men and women are socially impacted in term of quality of job, livelihood, gender equality and inclusion considering the gender relations previously mentioned. Then adding the category of education second-level question is how are educated and non-educated women and men socially impacted, and the third level will be how are educated and non-educated refuges/migrants’ women and men impacted socially. Following this method, we identified the most vulnerable group across these intersections in the comparison between countries and circular strategies*

**About survey questions and Likert scale conversions:**

We used existing frameworks to build the SIAF-CE. These pre-existing frameworks and tools suggested likert type questions for most of their indicators. So first, we took those questions and adapted to fit our research constraints when needed. The questions took workers' perspectives and assessed the level of occurrence of each attribute. For each formulated indicator, there were three questions. In the case of the OECD, they were originally framed in a 5-point Likert scale, so, to avoid a neutral answer, all Likert scales were converted to a 4-points scale. Then following the method of Suarez-Visbal et al. (2022), we normalised the selected frameworks to the same scale and score system. This resulted in a worker’s survey with five open-ended questions and 85 closed-end questions. The list of questions can be found in annex 2. The survey was pre-tested with 5 respondents in each country and then adjusted as needed, also workers surveys were tested on reliability through a Cronbach's alpha analysis. Finally, composite indicators were calculated for each answer using the average of the values. If the result was not an integer number, it was normalised by approximating the closest integer. In this way, we converted the values into the same qualitative scale.
