# Supplementary material for: The Social Impacts of Circular Strategies in the Apparel Value Chain; a Comparative Study Between Three Countries
Source: Circ Econ Sustain. 2022 Sep 6:1–34. Online ahead of print. doi: 10.1007/s43615-022-00203-8 (PMC9446664; doi:10.1007/s43615-022-00203-8)
Supplement: Supplementary file 3 — Supplementary file3: Annex 3. Survey SIAF-CE⚥ (DOCX 20.4 KB) [file 43615_2022_203_MOESM3_ESM.docx]

**Annex 3. Survey *SIAF-CE*⚥**

**Quality of Jobs (QOJ) Questionary**

| Indicator | Attributes | Questions |
| --- | --- | --- |
| Earning Quality | Gross income/hour | 1A1. What is your approximate salary per month (gross salary) (answer can be rate per hour/ month/ yearly) |
|  | Distribution of earnings with all workers (earning inequality | N/A |
| Labour Market Security | Risk of unemployment (risk and expected duration ) | To what extent do you agree with this statement: "I am worried I will lose my jobs in six months?" |
|  | Unemployment insurance (the coverage of the benefits and their generosity) | To what extent do you agree with this statement. "If I lose my job now I can apply for unemployment insurance?" |
| Work Environment | Job demands /Time pressure at work | To what extent do you agree with this statement: "My job involves working to tight deadlines" |
|  |  | To what extent do you agree with this statement: "My job involves working at very high speed" |
|  | Physical, health and Social factors | "At work I am exposed to handling or being in skin contact with chemical substances" (important for workers working on recycling or manufacturing mainly) |
|  |  | "My main job involves working in tiring or painful positions" |
|  |  | To what extent do you agree with this statement: "I get support and help from colleagues and co-workers when needed" |
|  |  | To what extent do you agree with this statement. "In general my immediate manager/supervisor respects me as a person" . IF SELF EMPLOYED ASK "in general I feel my work is valued by my clients" |
|  | Autonomy and learning opportunities | To what extent do you agree with this statement. "I think that my prospect for job opportunities are better because of training opportunities I receive at my work " (IF SELF EMPLOYED DON"T ASK THIS QUESTION) |
|  |  | To what extent do you agree with this statement: "I have enough opportunities to use my knowledge and skills in my current job" |
|  |  | To what extent do you agree with this statement.: "My job offers good prospects for career advancement" |
|  | Workplace relationship | To what extent do you agree with this statement: "I can influence decisions that are important for my work" |
|  |  | Would you say that the value of your work is properly recognised . IF SELF EMPLOYED YOU ALREADY ASKED THIS QUESTION |
|  | Work time arrangements/ working very long hours | To what extent do you agree with this statement: "For me, arranging to take an hour of two off during working hours to take care of personal patters is difficult" |
|  |  | How often are you asked to work on your free time to meet work demands? IF SELF-EMPLOYED HOW OFTEN DO YOU WORK ON FREE TIME OR WEEKENDS |

**Sustainable Livelihood (SL) Questions**

| Human Assets | Level of perceived health | How healthy do you believe you and your family are in general? |
| --- | --- | --- |
|  | Ability to work and retained work | Has any member of your family been unemployed for the last 2 years? (explain) |
|  | Level of education and or skills training of household members | Please select best option that describe your current situation. Would you say that other members of your household are….: (explain) |
| Natural Access | Access to natural Resources | With which of the following statement you identified the most? What natural resources you have access to close by (more than one option is possible) |
|  | Environmental Quality | With which of the following statement you identified the most? The waste and recycling service in my home area is: |
|  |  | Supplement: As a result of their job, how has the awareness and behaviour have change, do they recycle more, what has changed? |
|  |  | How well do you agree with this statement: I think my community is clean and properly maintained |
| Physical Assets | Access to good Housing | How well do you agree with this statement: "My current housing offers all the amenities we need as a family (amenities such as reliable electricity/gas/drinking water, separated living and sleeping spaces, internet) |
|  | access to transportation services and proximity of food sources | What kind of transportation do you have available in your community to go to work ? |
|  | Access to child/elderly care or recreation facilities | What kind of childcare and or elderly care facilities you have around your community? |
|  |  | Do you have access to a close by community centre, sport or playground? |
| Social Assets | Support from family and friends | With which statement you identified the most?. Do you get to spend time with your family? |
|  |  | With which statement you identified the most? |
|  | participation in community life | Does your family use any free programs offered in your community (if so which programs?) |
|  |  | Do members of your family are active members/regular volunteers of any social, environmental or political organization in your community |
| Financial Assets | Income/savings | With which of the following statement you identified the most? Is your (family) income enough to pay your family monthly expenses? |
|  | Money management (debts) | How well do you agree with this agreement: "We have a significant amount of debt in our household" (explain your answer) |
|  |  | How well do you agree with this agreement: We cannot save because all the money goes to paying debts |
|  | Possession of goods | Does your family owns any of the following assets (more than one option is possible) |
|  | circular Attributes | In which way you believe this job has directly contribute to your family access to any of the above five assets, (please select all that apply) |

**Gender Equality & Inclusion (GE&I) Questions**

| Economic Opportunity | Access to equitable safe and reliable employment | Do you agree with the following statement: "I am frequently under stress because I don’t have enough money to cover my family’s basic needs and save for emergencies" |
| --- | --- | --- |
|  |  | Do you agree with the following statement: "In my company women have the same job opportunities as men have". IF SELF EMPLOYED ASK "My kind of job can be equally performed by a man or a woman" |
|  |  | Within the factory/company, how do people OFTEN hear about job opportunities? [If no answer, give certain examples such as listed on a billboard or announced on the speaker.] |
|  |  | How is the starting salary for all positions OFTEN communicated? Is it told to the workers when they apply for a position? Or are the salaries posted somewhere ? if SELF EMPLOYED ASK IF YOU CAN MANAGE TO NEGOTIATE SAME HOURLY RATE WITH ALL CLIENTS |
| Access to & Control over Resource | Access to resource | How many productive assets do you have on your own name ?___# of assets (land, house, car, moto, computer, savings ( if applicable was this negotiated with your partner, please explain |
|  |  | Do you have an individual account in a bank or other financial institution? |
|  | Control over resources | When you are paid, do you decide what to do with your earnings? |
| Leadership & Training | Access to training & development of leadership skills | Do you agree with the following statement: in my company women have the same training and leadership/promotion opportunities at work as men have |
|  |  | Do you agree with the following statement: "I lack the abilities and skills to advance within the factory" |
|  |  | Do you agree with the following statement: "I think management supports the advancement of women workers in the factory" IF SELF EMPLOYED ask DO you support the advancement of women workers in the factory" |
| Voice & Collective Bargain | Voice & Collective bargain | Please select the best answer. Can you name the person(s) who are part of the worker committees and/or trade unions IF SELF EMPLOYED ASK ARE YOU MEMBER OF ANY WORKERS ORGANIZATION |
|  |  | In your company; would you say that the voice of women workers is effectively heard? |
|  |  | Do you agree with the following statement: "At my workplace, management does not take action on workers" feedback" |
| Violence & Harassment | Procedure knowledge | Is there a policy on violence and harassment in your factory? |
|  |  | Do you agree with the following statement: "If I wanted to report a case of violence or harassment in the workplace,( or community) I would know who or where to report it" |
|  |  | Since starting your job in this factory/company, have you ever refrained from using the toilet facility because someone prevented you from going, it was difficult to access the facility, or the toilets were not in proper condition for you to use them? |
|  | Norms & Culture | In the community where you live, is it common or acceptable for women to voice their opinion at home? |
|  |  | Do you agree with the following statement: "If a woman has been abused and reports it, she will bring shame to her family" |
| Health & Security | Access to healthcare | Do you have access to health services when you need them? |
|  |  | Do you agree with the following statement: "I am confident my employer is/would adjust tasks and/or provide protective equipment for pregnant workers" |
|  | Risk of accidents | Does your employer give you the necessary gear to perform your job or you have to get it yourself( if needed?)? ( if not needed go to question 6C4) if yes go to 6C5 |
|  |  | In the last 12 months have you had any accidents in the job, what is their cause? |
|  |  | Have you developed any respiratory/health problem since working here (relevant for manufacturing or recycling jobs only) |
| Sexual & Reproductive Health & Rights | Sexual and Reproductive Health and Rights | Do you agree with the following statement: "Women and men should share responsibility for childcare and housework" |
|  |  | Do you agree with the following statement: "My working hours allow me to perform my family and household duties" |
