# Supplementary material for: The Social Impacts of Circular Strategies in the Apparel Value Chain; a Comparative Study Between Three Countries
Source: Circ Econ Sustain. 2022 Sep 6:1–34. Online ahead of print. doi: 10.1007/s43615-022-00203-8 (PMC9446664; doi:10.1007/s43615-022-00203-8)
Supplement: Supplementary file 4 — Supplementary file4: Annex 4. Circular Jobs Inventory and gender baseline (DOCX 156 KB) [file 43615_2022_203_MOESM4_ESM.docx]

**Annex 4. Circular Jobs Inventory and gender baseline**

**The Netherlands**

| CIRCULAR STRATEGY | | JOBS | JOB CHARACTERISTICS | | | SOCIO-DEMOGRAPHICS/GENDER BASELINE | | | | | | |
| --- | --- | --- | --- | --- | --- | --- | --- | --- | --- | --- | --- | --- |
|  |  |  | **TASKS** | **SKILLS** | **CONTRACT type/hours** | **EDUCATION** | **GENDER** | **AGE** | **MARITAL STATUS** | **MIGRATION** | **CHILDREN** |  |
| REPAIR  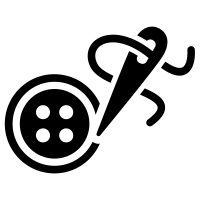 | **in-house** | **Manager** | - Tailoring  - Business administration  - Sales  - Managing orders  - Website management  - Staff safety & well-being | - Tailoring  - Design  - Material knowledge  - Social skills | 50% permanent  50% self-employed  50% full-time  50% overwork | 100% technical degree | 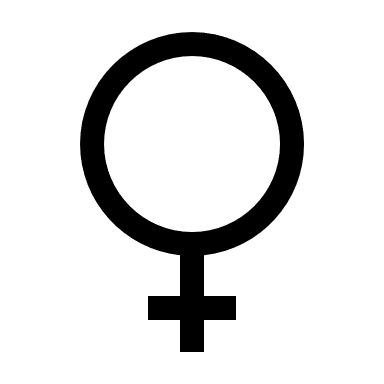100%  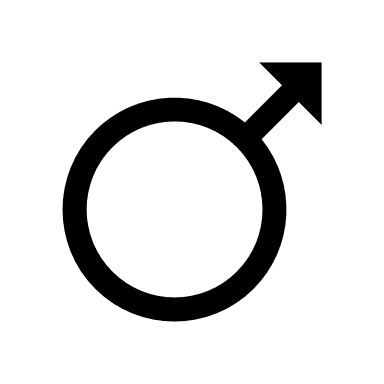0% | 100% 19-35 years | 100% single | 0 % | 100% no children |  |
|  |  | **Sales assistant** | - Client support  - Repairs - Donation management | - Communication, - Sales  - Sewing  - Creativity  - Openness | 75% permanent  25% short term  75% part-time 25% full-time | 50% technical degree  50% university | 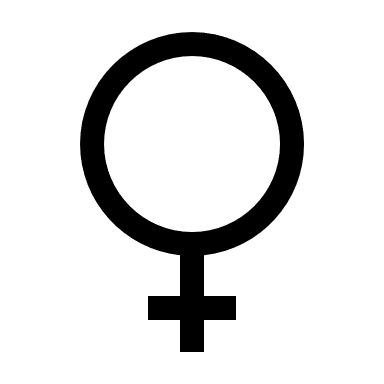50%  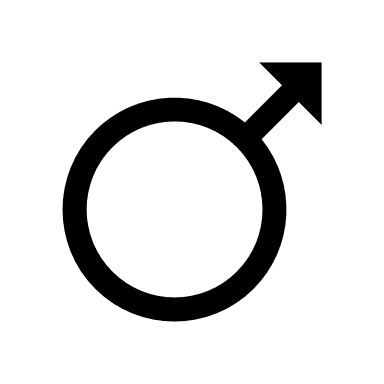50% | 100% 19-35 | 100% single | 25 %  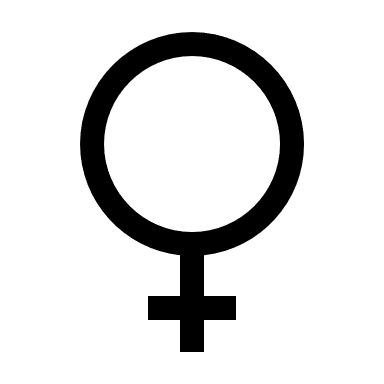100%  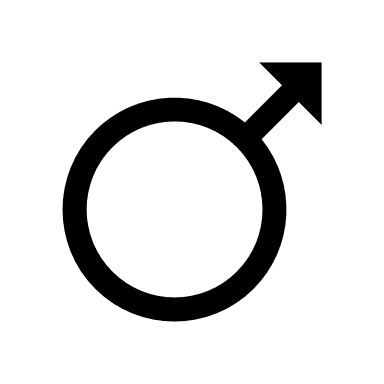0% | 100% no children |  |
|  | **independent** | **Tailor** | - Design products  - Teaching sewing  - Costumer support  - Administration | - Sewing  - Communication | 100% one year  33% part-time  66% overwork | 33% primary school  33% technical degree  33% university | 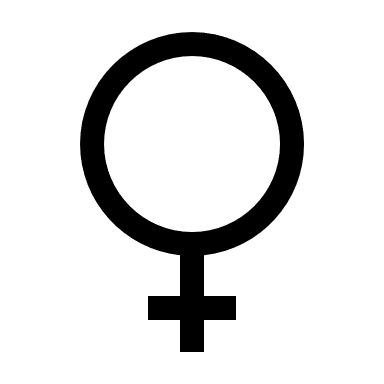33%  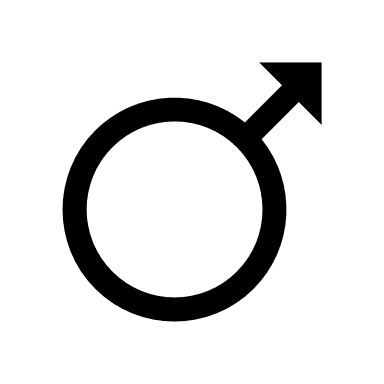66% | 33% 51-65  33% 65+  33% no response | 33% single with children  33% married without children  33% other | 67 %  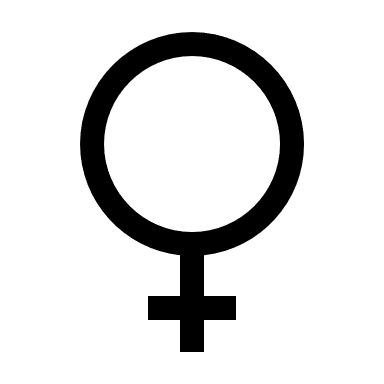0%  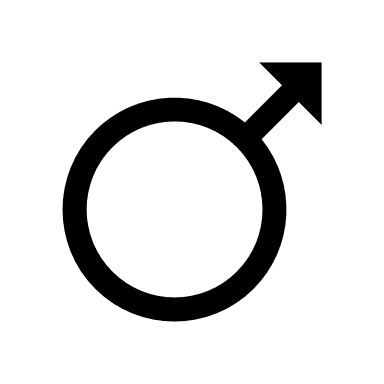100% | 66% less than 3  33% less than 5 |  |
| RENTAL 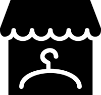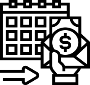 | **B2C** | **Manager** | - Costumer support  - Business planning  - Administration  - Website management | - Creativity  - Persistence  - Collaborative  - Organization | 100% self-employed  80% part-time  20% overwork | 100% university | 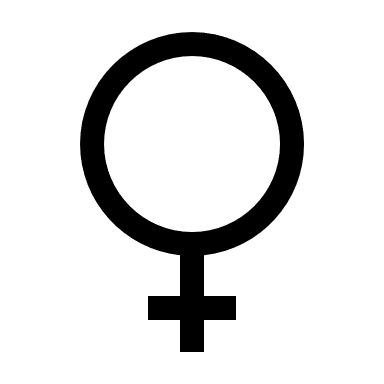60%  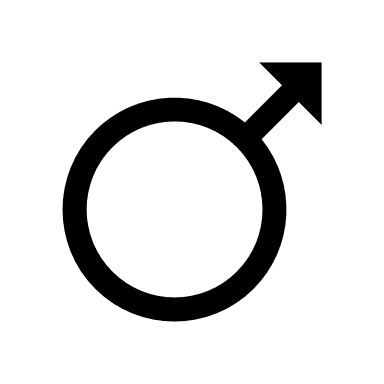40% | 40% 19-35  40% 36-50  20% 51-65 | 40% married without children  60% married with children | 20 %  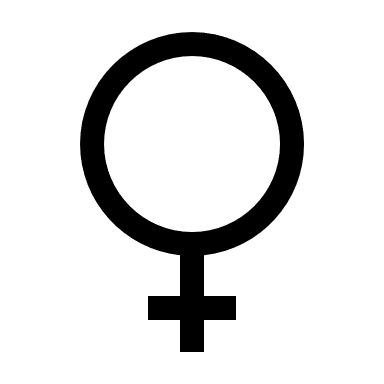33%  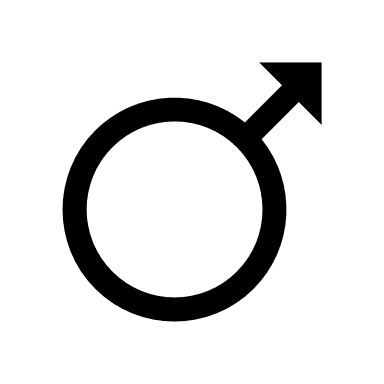0% | 20% no children  40% one child  40% less than 3 |  |
|  | **B2B** | **Manager** | - Scanning  - Refilling | - Communication  - Team player  - Hardworking  - Leadership | 100% permanent  100% full-time | 100% technical degree | 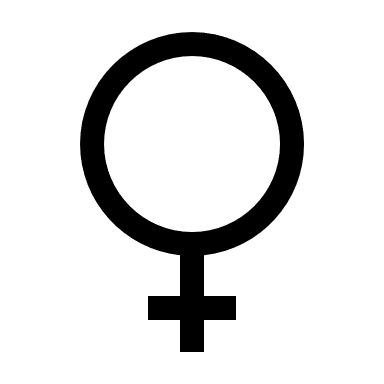0%  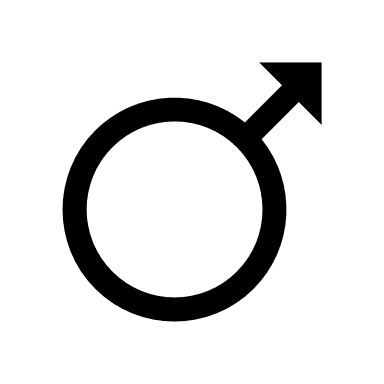100% | 100% 36-50 | 100% married with children | 0 % | 100% less than 3 |  |
|  |  | **Designer** | - Design  - Training | - Creative  - Open-minded  - Team player  - Technical skills | 100% temporary  100% part-time | 100% technical degree | 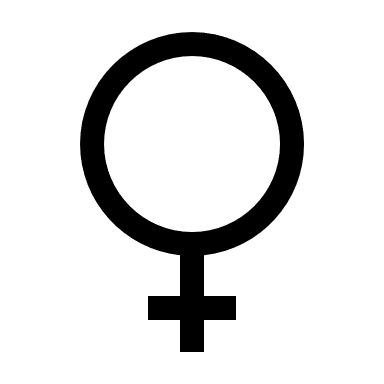100%  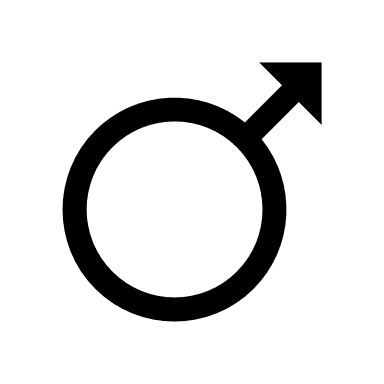0% | 100% 36-50 | 100% married with children | 0 % | 100% no children |  |
|  |  | **Logistic clerk** | - Order  - Scanning  - Inventory | - Scanning  - Communications | 100% one year  100% full-time | 100% university | 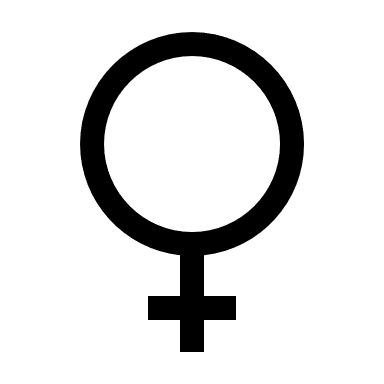0%  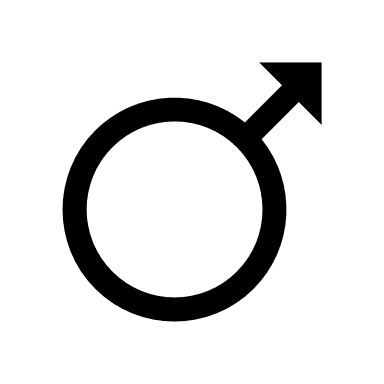100% | 50% 19-35  50% 36-50 | 80% married with children | 50 %  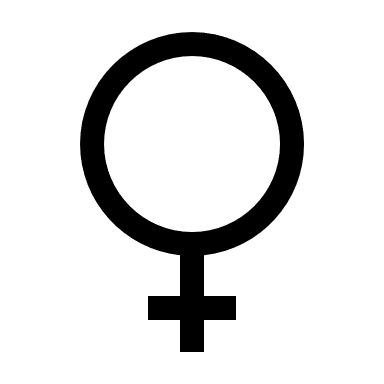0%  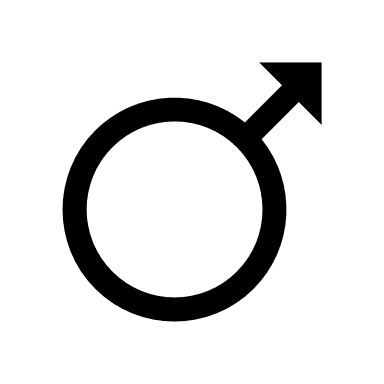100% | 100% no children |  |
| RESALE – local  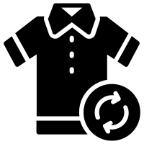 | | **Manager** | - Creative business planning  - Social networking  - Marketing & sales  - Buying  - Training  - Administration  - Cleaning | - Creativity  - Aesthetics  - Business skills  - Passion  - Resilience  - Detail oriented | 29% other  71% temporary  43% full-time  43% part-time  14% overwork | 14% primary school  72% technical degree  14% university | 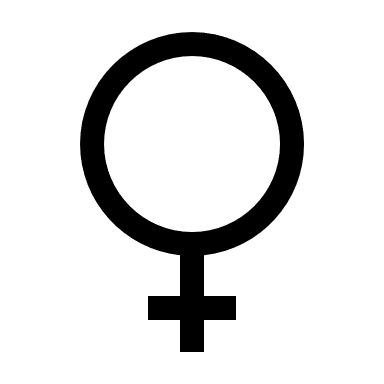86%  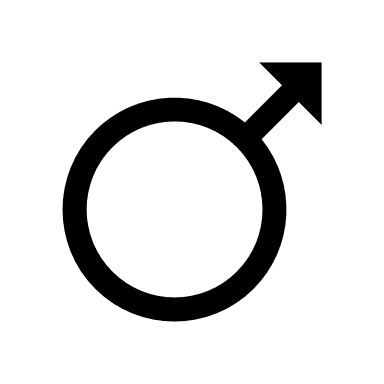14% | 14% 19-35  43% 36-50 | 14% married with no children  42% married with children | 28 %  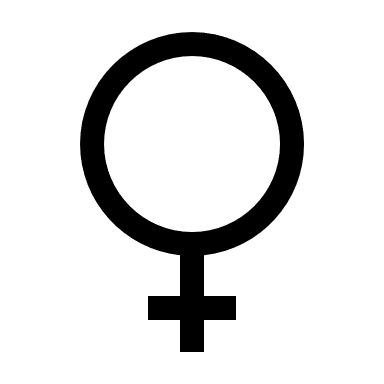50%  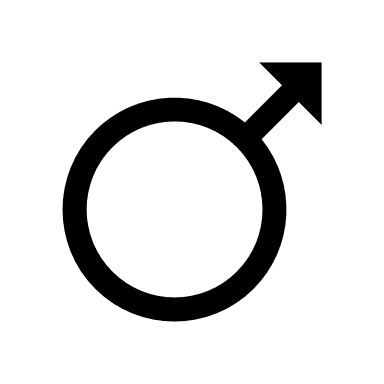50% | 14% no children  14% one child  28% less than 3  14% other |  |
|  |  | **Sales Assistant** | - Sales | - Stress management  - Empathy  - Selling skills  - Communication  - Hard-working  - Organization | 33% short term 33% one year 33% permanent  100% part-time | 33% technical degree  66% university | 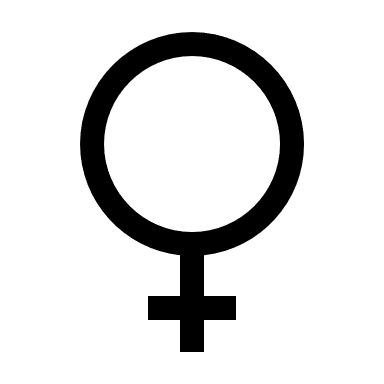66%  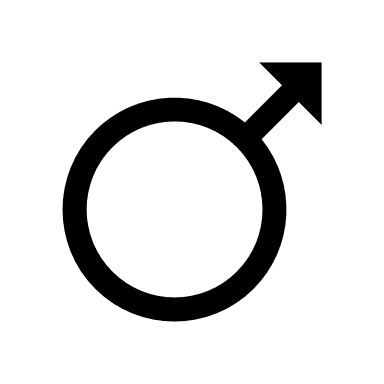33% | 66% 19-35 | 85% married with no children | 67 %  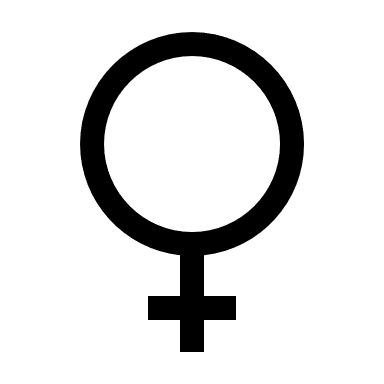100%  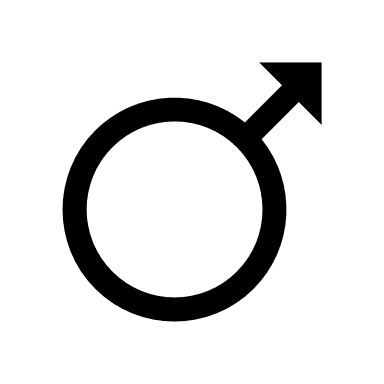0% | 100% no children |  |
| REMANUFACTURE  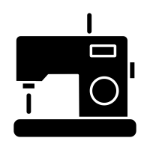 | | **Designer** | - Organization  - Management  - Circularity  - Sourcing  - Production | - Problem solving  - Resilience  - Flexibility  - Proactivity  - Materials knowledge | 50% permanent 50% short term  100% full-time | 50% technical degree  50% university | 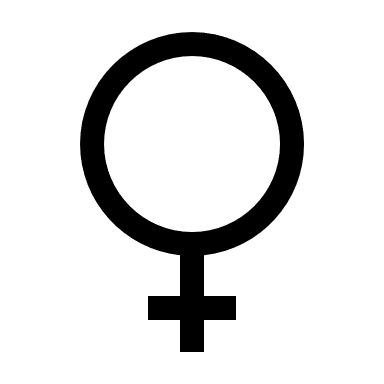100%  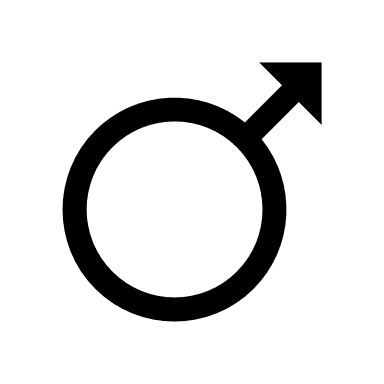0% | 50% 19-35  50% 51-65 | 75% married without children | 0 % | 50% no children  50% less than 3 |  |
|  |  | **Tailor/ Sewing Machine Operator** | - Tailoring  - Sewing  - Recycling  - Maintenance of machines  - Pattern printing  - Managing production | - Tailoring  - Sewing  - Communication  - Willingness to learn  - Patience  - Handiness  - Humbleness | 43% short term 43% one year  5% other  81% part-time 19% full-time | 29% primary school  38% secondary school  19% technical degree | 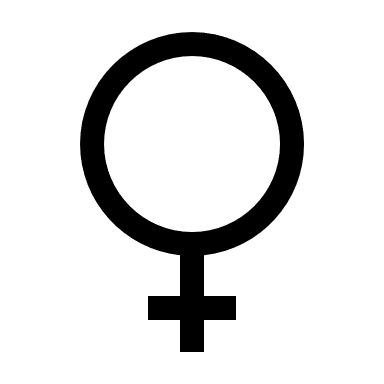76%  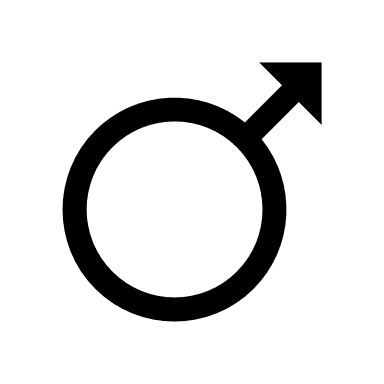24% | 57% 36-50 | 47% married with children  33% divorced with children | 71 %  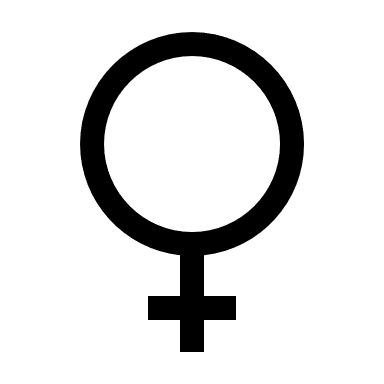87%  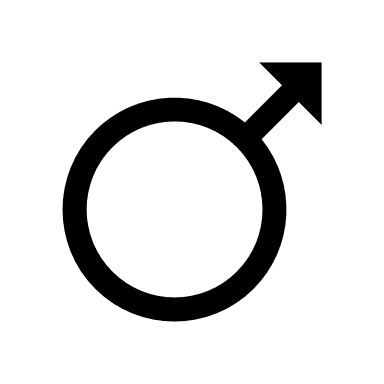13% | 24% no child  24% one child  29% less than 3  24% less than 5 |  |
| RECYCLE  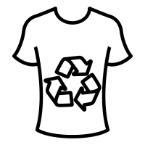 | | **Manager** | - Operation management | - Communication  - Multitasking | 100% permanent  100% full-time | 100% technical degree | 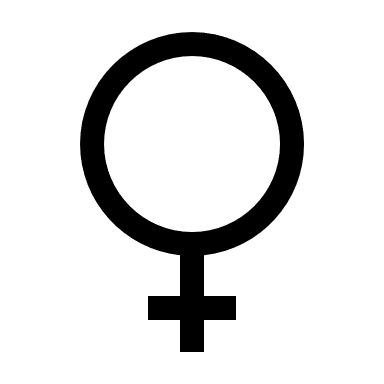0%  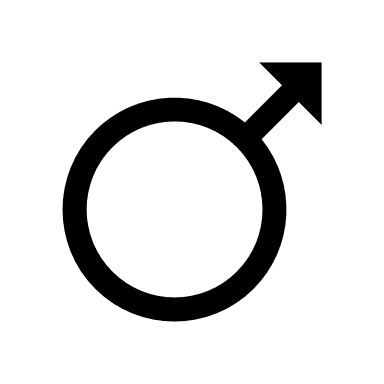100% | 100% 51-65 | 100% married with children | 0 % | 100% less than 3 |  |
|  |  | **Buyer** | - Buying unsorted clothes  - Establish partnerships | - Analytical skills  - Logical thinking and understand trends, quality of clothing, value of brands, quality of textile | 100% permanent  100% part-time | 100% university | 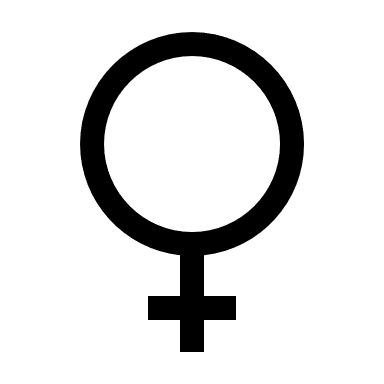100%  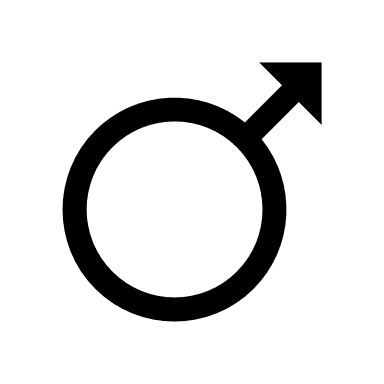0% | 100% 19-35 | 100% single | 0 % | 60% no children  20% one child  20% less than 3 |  |
|  |  | **Logistic Clerk/Sorter** | - Unload containers  - Driving trucks  - Sorting  - Trainings  - Visual merchandising  - Logistics | - Communication  - Being careful  - Patience  - Knowledge of materials  - Operating machines | 56% permanent  22% one year  11% short term  67% full-time  33% part-time | 22% secondary school  56% technical degree  22% university | 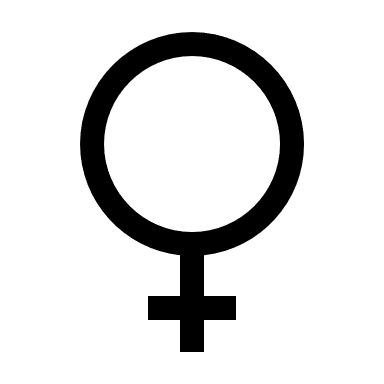67%  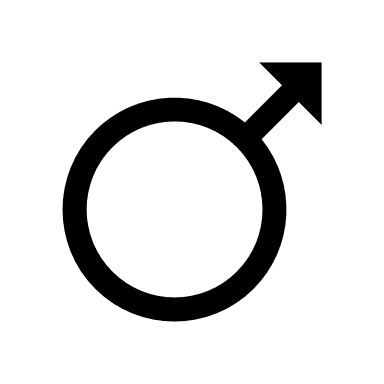33% | 33% 19-35  33% 36-50  33% 51-65 | 11% single  22% single with children  44% married with children  22% divorced with children | 56%  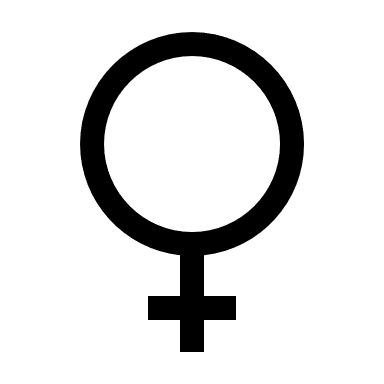60%  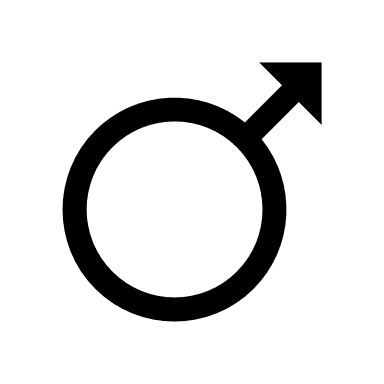40% | 56% no children  11% one child  33% less than 3 |  |

**Spain**

| CIRCULAR STRATEGY | JOBS | JOB CHARACTERISTICS | | | SOCIO-DEMOGRAPHICS/GENDER BASELINE | | | | | |
| --- | --- | --- | --- | --- | --- | --- | --- | --- | --- | --- |
|  |  | **TASKS** | **SKILLS** | **CONTRACT type/hours** | **EDUCATION** | **GENDER** | **AGE** | **MARITAL STATUS** | **MIGRATION** | **CHILDREN** |
| RENTAL  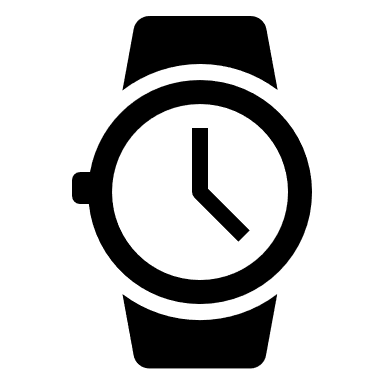 | **Manager** | - Creating brand awareness  - Costumer care  - Sales  - Team management  - Product control  - Finances management | - Leadership  - Team coordination  - Communication  - Social and environmental awareness  - Commitment  - Knowledge of the industry  - Negotiation skills  - Organizational skills  - Proactivity  - Thinking outside the box | 60% permanent  40% other  60% overwork  20% part-time | 60% university  40% other | 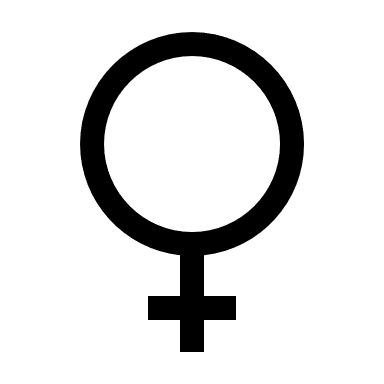60%  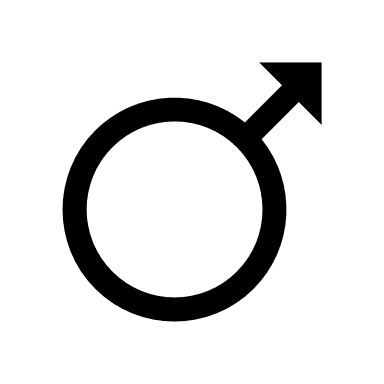40% | 80% 19-35  20% 36-50 | 80% single  20% married with children | 0% | 60% no children  20% less than 3 |
| 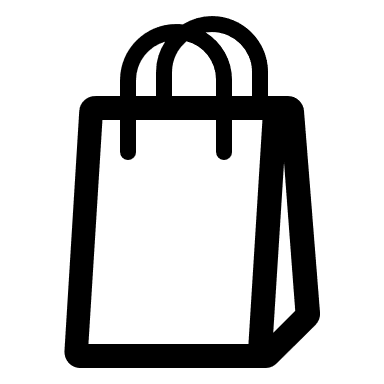RESALE – local | **Manager** | - Organize the international marketing team  - Plan, review and schedule newsletters and publication of clothing  - SEO marketing  - Coordination | - Organization  - Responsibility  - Management skills and empathy  - Administration and extensive legal-legislative knowledge  - HR  - Good communication  - Methodical  - Proactiveness | 100% permanent  100% fulltime | 100% other | 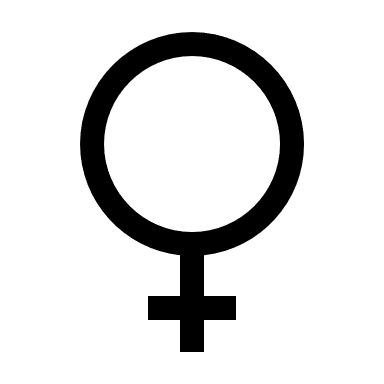100%  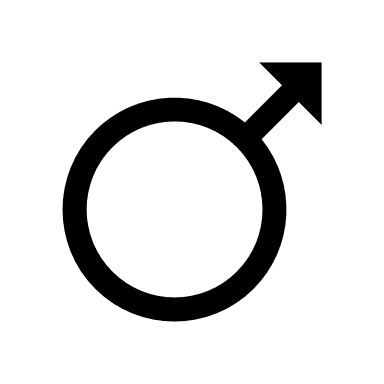0% | 50% 19-35  50% 36-50 | 100% married without children | 0% |  |
|  | **Project Manager** | - Analysis of legislation & support for cooperative projects  - Preparation of technical reports  - Representation in national and international industry forums  - Process optimization  - Follow-up of activities and team coordination  - Communication  - Technical and economic follow-up of projects | - Analytical skills  - Scientific-technical knowledge of law and legislation  - Environmental knowledge  - Management skills  - Creativity  - Writing and communication skills  - Financial control  - Social and environmental knowledge  - Project coordination | 33% permanent  66% other  100% full-time | 100% university | 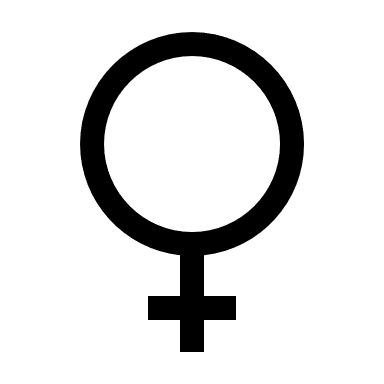33%  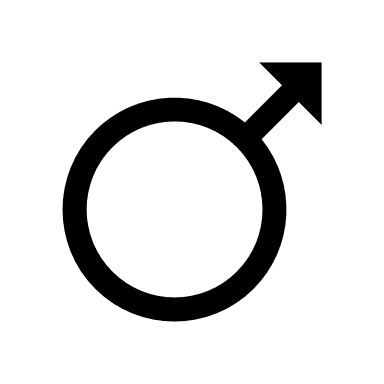66% | 100% 36-50 | 33% married without children  33% married with children  33% divorced with children | 0% | 33% no children  33% 1 child  33% less than 3 |
|  | **Designer** | - Designing the ad for the Google Ads platform | - Curiosity  - Creativity | 100% for 1 year  100% full-time | 100% university | 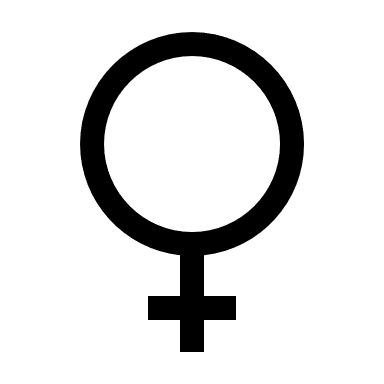0%  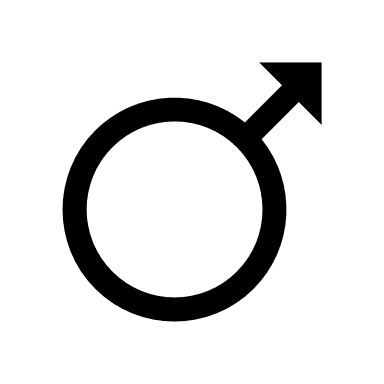100% | 100% 19-35 | 100% single | 100 %  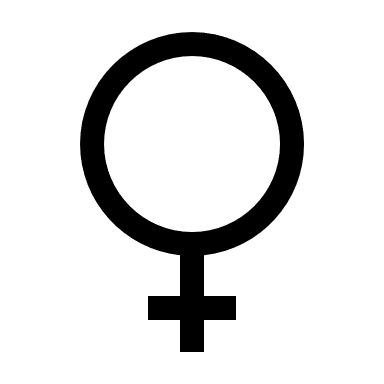0%  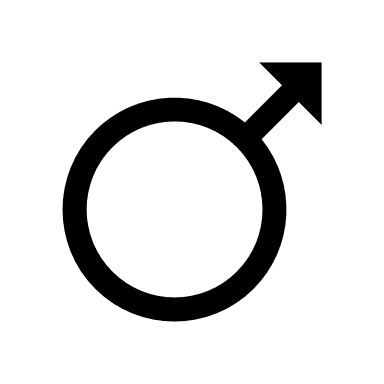100% |  |
|  | **Marketing** | - Design, coordinate and execute external and internal communication and marketing strategies  - Elaboration of the graphic environment of the fashion stores  - Brand image, influencer marketing, social networks management | - Coordination and management capacity  - Communication  - Proactivity  - Organization | 66% permanent  33% for 1 year  100% full-time | 100% university | 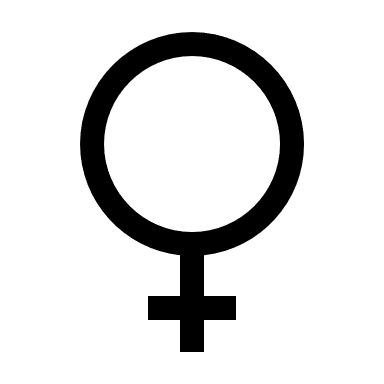100%  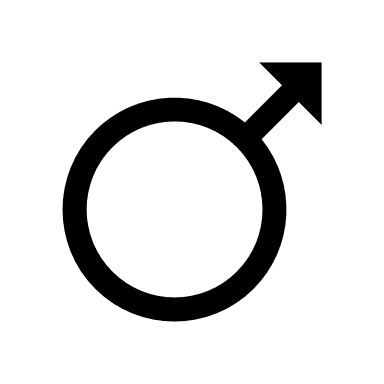0% | 100% 19-35 | 100% single | 33 %  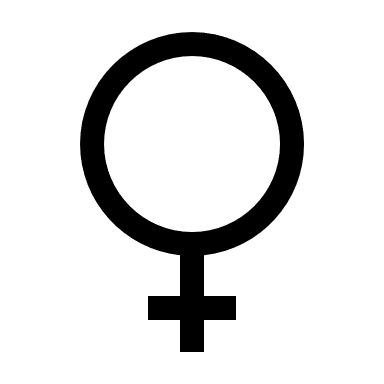100%  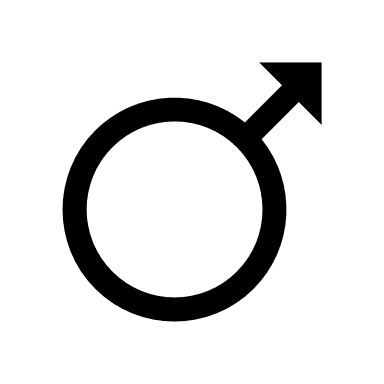0% | 66% no children |
|  | **Sales Assistant** | - Customer service  - Partnership management  - Preparation of newsletters  - Translation  - Creating email marketing campaigns  - Finding and capturing influencers and celebrities  - Supporting the team  - Analysing data from sales campaigns  - Making sales campaigns  - Managing all social networks | - Ability to perform many tasks  - Organized in your work  - Ability to work autonomously  - Rigorous organization  - Computer skills  - Knowledge of current fashion  - Knowledge of digital marketing | 66% for 1 year  33% other  100% part-time | 66% university  33% other | 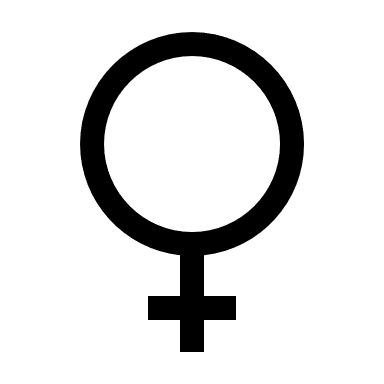100%  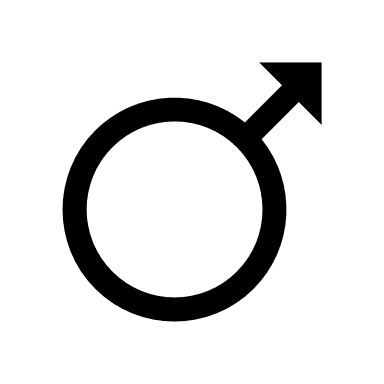0% | 100% 19-35 | 66% single | 66 %  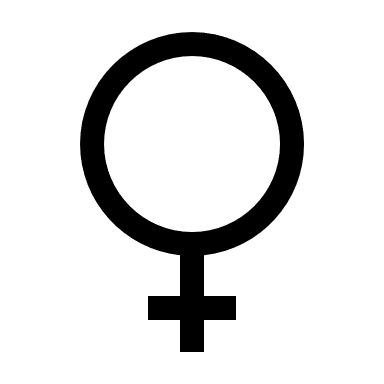100%  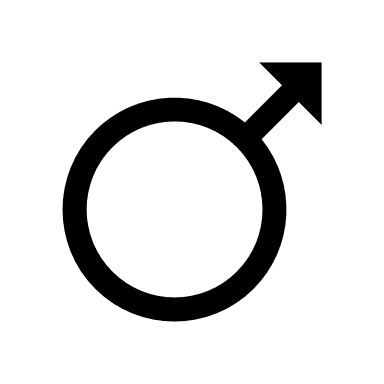0% | 66% no children |
| REMANUFACTURE  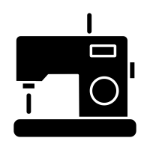 | **Manager** | - Creative direction  - Negotiation with clients  - Production and supplier management  - Managing the creative team  - Manage the digital communication of the brand | - Communication  - Creativity  - Interpersonal skills  - Knowledge  - Experience  - Vision for the future | 100% permanent  66% full-time  33% overwork | 66% other  33% university | 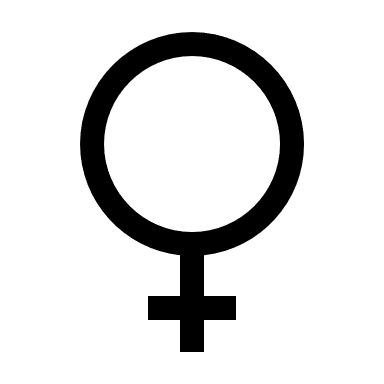33%  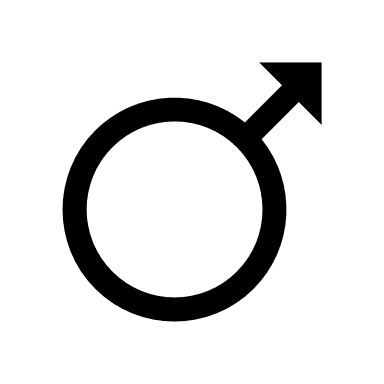66% | 66% 19-35 | 66% single  33% married without children | 0% | 66% no children |
|  | **Project Manager** | - Taking care of the direction of the collection, prints and trends | - Aesthetic taste  - Drawing  - Awareness  - Empathy  - Patience | 100% permanent  100% fulltime | 100% university | 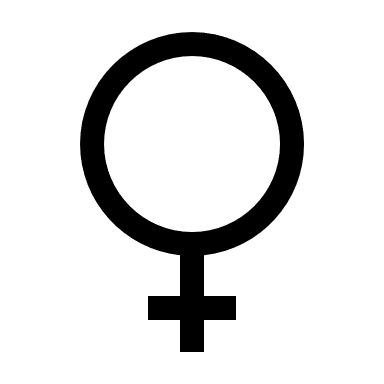0%  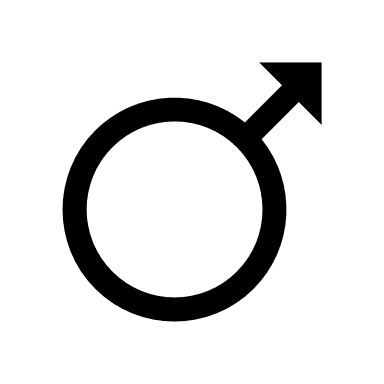100% | 100% 19-35 | 100% married without children | 0% |  |
|  | **Designer** | - Content creation  - Social media management  - Collaborations with external agents  - Graphic design  - Marketing and branding  - Product development  - After-sales support  - Art direction  - Clothing design  - Market research  - Search for suppliers  - Search for more "eco-friendly" solutions  - Press and media relations | - Creativity  - ICT proficiency  - Language proficiency  - Teamwork  - Adaptability  - Graphic design tools  - Writing skills  - Decision-making  - Speed  - Confidence  - Communication  - Flexibility  - Empathy  - Organization  - Proactivity | 50% permanent  33% short-term  16% for 1 year  83% fulltime  16% part-time | 50% other  28% university  14% technical degree | 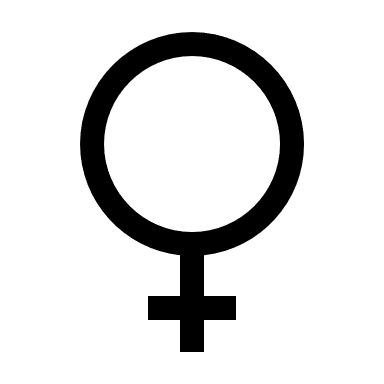100%  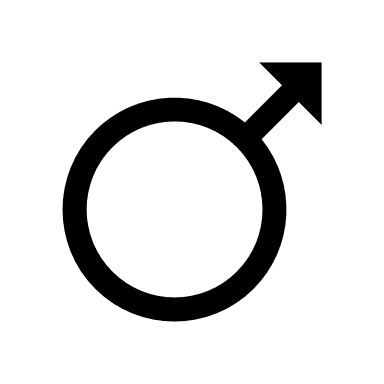0% | 100% 19-35 | 83% single  17% married without children | 0% | 100% no children |
|  | **Buyer** | - Stock control  - Order preparation  - Relationship with suppliers for production  - Commercial action in the sales channel to stores and B2B | - Organization skills  - Interpersonal skills | 100% other  100% fulltime | 100% university | 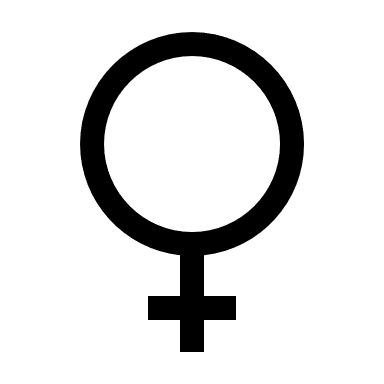100%  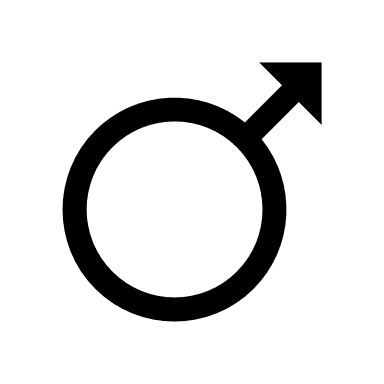0% | 100% 19-35 | 100% single | 0% |  |
|  | **Tailor** | - Cutting and sewing  - Garment making,  - Selecting the denim we recycle  - Weaving  - Pattern making  - Scaling the designs that are brought to us and making the prototypes before production | - Sewing  - Knowing the machine  - Adaptability  - Willingness to learn  - Patience  - Perfectionist  - Detail oriented  - Be very methodical  - Self-sufficient | 57% permanent  28% short-term  14% other  57% part-time  28% fulltime  14% overwork | 42% university  28% technical degree  14% secondary school  14% primary school | 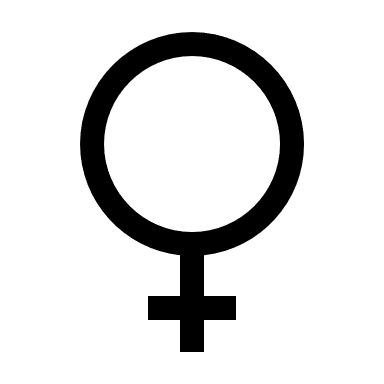85%  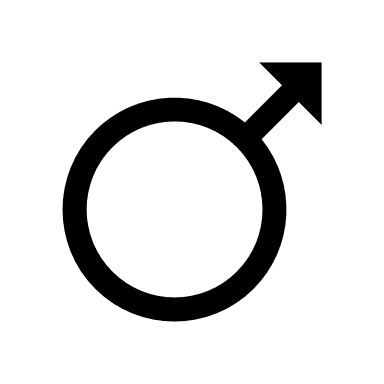15% | 71% 26-50  14% 19-35  14% 51-65 | 42% married with children  28% single with children  28% single | 42 %  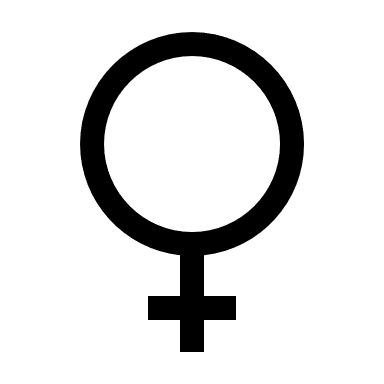100%  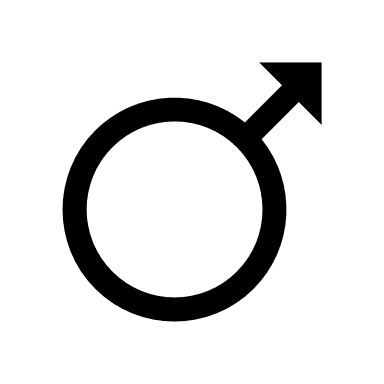0% | 57% no children  28% less than 3  14% less than 5 |
|  | **Sales Assistant** | - Supplier relations  - Showroom  - Talk to stylists  - Contact suppliers  - Catalogues  - Layout  - Social networks | - Be fast and practical  - Presenting skills  - Know languages  - Be able to manage social networks | 100% other  100% fulltime | 100% university | 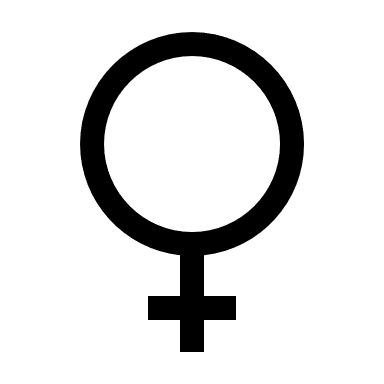100%  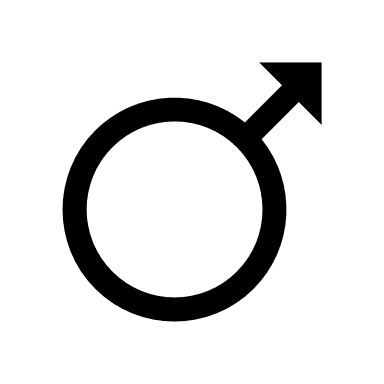0% | 100% 19-35 | 100% single | 0% | 100% no children |
| RECYCLE  (use of recycled material)  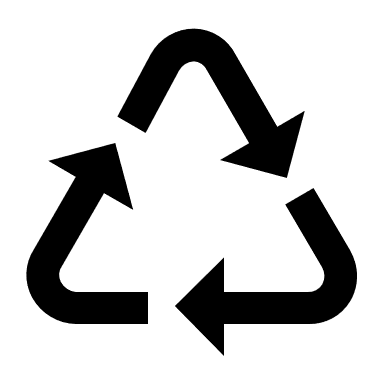 | **Manager** | - Traceability of the production sites  - Social and environmental assessment  - Non-financial reporting of the company | - Social and environmental skills  - Knowing the centre of production and the product | 100% permanent  100% full-time | 100% technical degree | 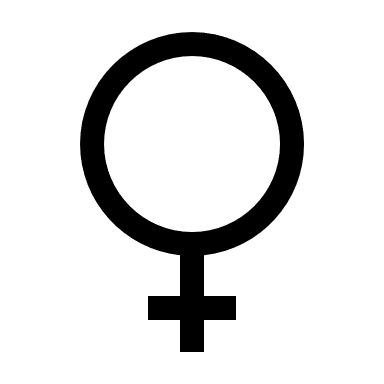100%  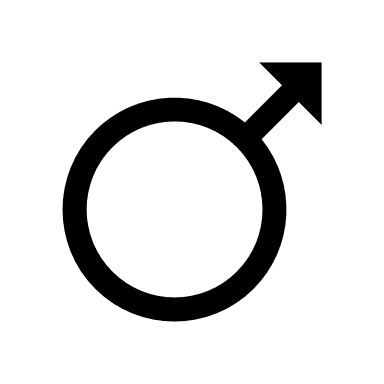0% | 100% 36-50 | 100% married with children | 0% | 100% 1 child |
|  | **Designer** | - Search for inspiration to create jewellery collections  - Home decoration  - Contact with suppliers  - Development and modification of all phases of product design  - Plan  - Manage  - Control  - Design garments  - Prints and graphics | - Organizational skills  - Agility  - Meticulousness  - Rigorousness  - Drawing  - Design and use of design programs  - Creativity  - Adaptability  - Time management | 50% permanent  25% temporary with an agency  25% other  75% full-time | 75% university  25% other | 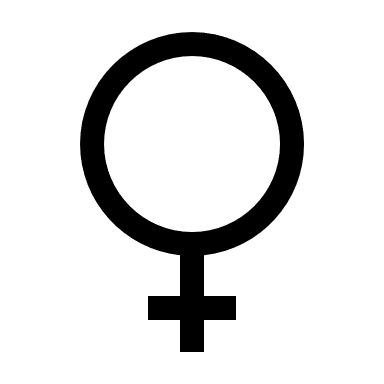100%  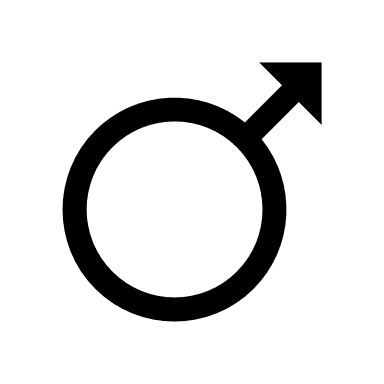0% | 75% 19-35  25% 36-50 | 50% single  25% married without children  25% married with children | 0% | 25% no children |
|  | **Buyer** | - Warehouse and product preparation  - Management of the collection development process,  - Planning and management of the collection purchasing process  - Team management  - Analysis of results  - Purchasing management and negotiations  - Sourcing planning  - Strategic planning  - Merchandising  - Sales strategy  - Point-of-sale training | - Analytical skills  - Communication/people skills  - Planning  - Strategy  - Knowledge of supply chain  - Existing sustainability tools  - Legislation  - Quality  - Negotiation skills  - Product and market knowledge  - Languages  - Accuracy  - Agility  - Teamworking | 60% permanent  40% other  40% full-time  60% other | 20% technical degree  20% university  20% other | 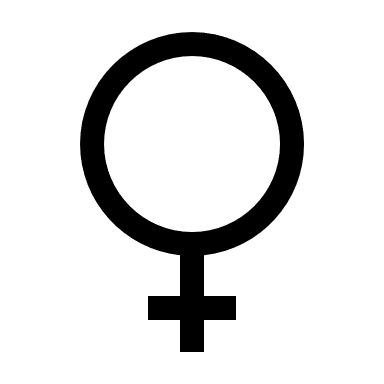100%  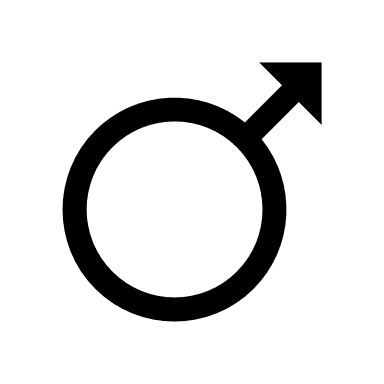0% | 100% 36-50 | 40% married without children  20% married with children | 20%  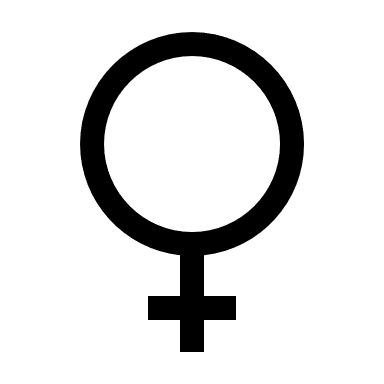100%  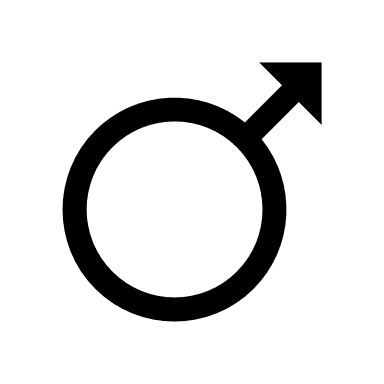0% | 20% no children  20% 1 children  20% less than 3 |
|  | **Administration** | - Administration and finances | - Know-how and focus | 100% permanent | 100% technical degree | 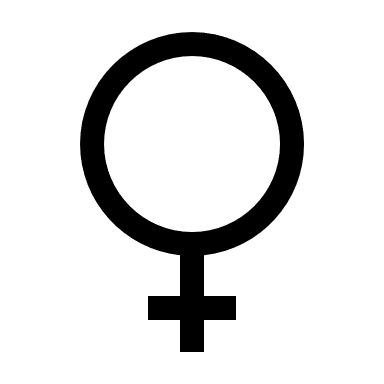0%  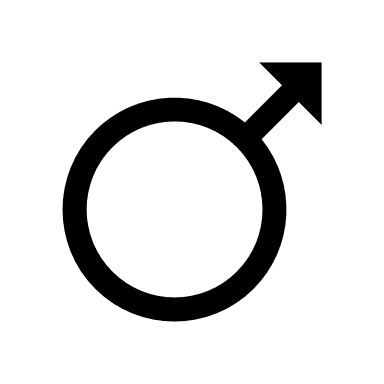100% | 100% 36-50 |  | 0 % |  |
|  | **Logistic clerk** | - SAP master data maintenance | - Knowing SAP software | 100% permanent | 100% university | 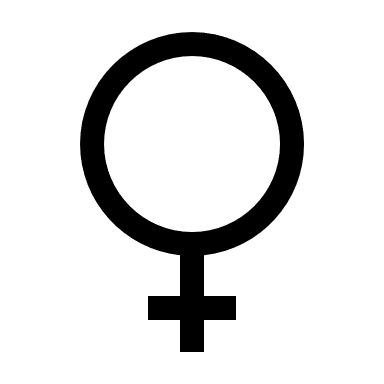100%  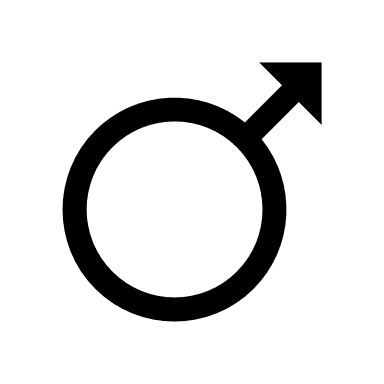0% | 100% 36-50 | 100% single with children | 0% | 100% 1 child |

**India**

| CIRCULAR STRATEGY | JOBS | JOB CHARACTERISTICS | | | SOCIO-DEMOGRAPHICS/GENDER BASELINE | | | | | |
| --- | --- | --- | --- | --- | --- | --- | --- | --- | --- | --- |
|  |  | **TASKS** | **SKILLS** | **CONTRACT type/hours** | **EDUCATION** | **GENDER** | **AGE** | **MARITAL STATUS** | **MIGRATION** | **CHILDREN** |
| REPAIR  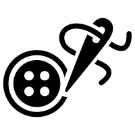 | **Tailor** | - Pattern making  - Cutting  - Stitching  - Altering old clothes  - Redesigning old clothes | - Stitching  - Cutting | 100% no contract  100% overwork | 50% primary school 50% secondary school | 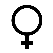0%  100% | 50% 19-35 50% 36-50 | 100% married or de facto with children | 0% | 100% less than 5 |
|  | **Sewing Machine Operator** | - Altering  - Reshaping clothes | - Altering - Reshaping clothes | 100% no contract  100% part-time | 100% secondary school | 100%  0% | 100% 19-35 | 100% married or de facto with children | 0% | 50% 1 child 50% less than 3 children |
|  | **Dry Cleaner** | - Dry-cleaning - Freshening up old garments  to increase their life - Removing stains  - Repairing for any cuts or  tears | - Knowledge of  different garments  - Dry cleaning  - Repairing clothes | 100% no contract  100% overwork | 66% secondary school 33% university | 0%  100% | 66% 51-65 33% 36-50 | 100% married or de facto with children | 0% | 66% less than 3 33% less than 5 |
|  | **Repair Worker** | - Mending cloth by sewing holes or cuts - Repairing clothes for re-use | - Knowledge of repairing cloth | 50% no contract 50% self-employed  50% full time 50% overwork | 50% primary school 50% others (not educated) | 0%  100% | 100% 36-50 | 100% married or de facto with children | 0% | 100% less than 5 |
|  | **Cobbler** | - Mending shoes with holes or any kind of wear and tear  - Repairing shoes and stitching new ones | - Shoe-making | 100% self-employed  100% overwork | 50% primary school 50% others (not educated) | 0%  100% | 100% 36-50 | 100% married or de facto with children | 0% | 100% less than 5 |
| RENTAL | **Manager** | - Supervision  - Store management  - Customer service  - Warehouse management  - Maintaining inventory  - Overlooking dispatch at warehouse  - Operations and sales | - Management  - Supervision  - Leadership | 66% permanent 33% yearly contract  100% overwork | 100% university | 0%  100% | 100% 19-35 | 33% married or de facto with children 66% married or de facto with no children | 0% | 66% no child 33% one child |
|  | **HR Manager** | - Documentation  - Recruitment | - Human Resource  Management | 100% permanent  100% overwork | 100% university | 0%  100% | 100% 19-35 | 100% single | 0% | 100% no child |
|  | **Sales Assistant** | - Customer service  - Designing  - Visual merchandising  - Fashion consultancy  - Sales  - Billing | - Designing  - Sales  - Customer service | 100% permanent  100% overwork | 100% university | 33%  66% | 100% 19-35 | 66% single  33% married or de facto with no children | 0% | 100% no child |
|  | **Tailor** | - Tailoring  - Alterations and fitting  - Customer service | - Tailoring  - Alterations and fitting | 100% permanent  100% overwork | 100% secondary school | 0%  100% | 100% 19-35 | 100% married or de facto with children | 0% | 100% less than 3 children |
|  | **Logistics Clerk** | - Delivery personnel  - Pickup and drop deliveries | - Delivery  - Driving | 100% permanent  100% overwork | 100% secondary School | 0%  100% | 100% 19-35 | 100% married or de facto with children | 0% | 100% one child |
| RESALE | **Shopkeeper** | - Sales  - Procuring clothes  - Sorting | - Sales | 100% permanent  100% overwork | 50% secondary school, 50% other (no education) | 0%  100% | 50% 51-65 25% 36-50 25% 19-35 | 75% married or de facto with children 25% single | 0% | 75% less than 5 children 25% no child |
|  | **Sales Assistant** | - Sales  - Sorting  - Luxury sales management | - Ability to convince a customer  - Sales | 85% permanent 15% yearly contract  100% overwork | 71% university 29% secondary school | 15%  85% | 57% 19-35 43% 36-50 | 57% married or de facto with children 14% married or de facto with no children 28% single | 0% | 43% no child 14% one child 28% less than 3 children |
|  | **Independent Repairer** | - Buy used clothes  in exchange for utensils and then sell those goods in the resale market after repairing | - Persuasion  - Sales  - Buying and procurement  - Repair | 100% no contract  100% overwork | 100% other (uneducated) | 100%  0% | 80% 51-65 20% 36-50 | 100% married or de facto with children | 0% | 80% more than one child |
|  | **Technician** | - Quality Control  - Technical support  - Digital portal handling  - Digital marketing | - Knowledge of the product  - Technical knowledge | 50% permanent 50% yearly contract  100% overwork | 50% university 50% secondary school | 0%  100% | 100% 19-35 | 50% married or de facto with children 50% single | 0% | 50% no child 50% one child |
|  | **Logistics Clerk** | - Checking orders  - Review  - Packing  - Courier  - Disperse final products | - Reviewing  - Knowledge of product | 100% permanent  100% overwork | 50% technical degree 50% secondary school | 0%  100% | 100% 36-50 | 100% married or de facto with children | 0% | 100% less than 3 children |
| REMANUFACTURE | **Sewing Machine Operator** | - Stitching  - Cutting  - Handwork and embroidery | - Tailoring (stitching)  - Cutting  - Creativity  - Quick learner | 45% no contract 55% permanent  73% overwork 9% full time 18% part time | 64% secondary school 27% university 9% other (uneducated) | 82%  18% | 55% 19-35 36% 36-50 9% 51-65 | 64% married or de facto with children 36% single | 0% | 55% more than one child |
|  | **Manager** | - Accounting  - Supervising sales  - Administrative work  - Provide training  - Coordinate operations  - Supervise work | - Accounting  - Skill of sales and marketing  - Ability to supervise  - Leadership  - Managerial duties  - Stitching | 25% yearly contract 75% permanent  100% overwork | 25% university 75% secondary school | 75%  25% | 25% 19-35 75% 36-50 | 75% married or de facto with children 25% single | 0% | 25% no child 75% less than 3 children |
|  | **Technician** | - Packing  - Market work - Quality control  - Sourcing raw material  - Checking the quality of the final product | - Ability to check  quality  - Buying ability  - Good research ability  - Knowledge of material  - Knowledge of right prices | 33% permanent 33% yearly contract 33% other  100% overwork | 66% secondary school 33% university | 0%  100% | 66% 19-35 33% 36-50 | 100% married or de facto with children | 0% | 66% less than 3 children 33% less than 5 children |
|  | **Designer** | - Creative designing  - Public Relations  - Graphic designing  - Production and design | - Communication  - Technical design aspects  - Sampling  - Production  - Managing supply  - Merchandising | 100% permanent  100% over work | 100% university | 100%  0% | 100% 19-35 | 100% single | 0% | 100% no child |
|  | **Logistics Clerk** | - Provide assistance  in QC and tailoring | - Checking for quality and providing assistance where required | 100% permanent  100% overwork | 100% other (uneducated) | 0%  100% | 100% 36-50 | 100% married or de facto with children | 0% | 100% less than 5 children |
|  | **Tailor** | - Handwork and embroidery  - Cutting textile  - Dress and home decor making  - Pattern making  - Stitching  - Teaching the skill and supervising  - Making the carpet | - Tailoring  - Pattern making  - Stitching  - Cutting  - Ability to teach juniors | 50% others (no contract) 50% permanent  61% overwork 33% parttime 6% full time | 33% other (uneducated) 50% secondary school 11% university 6% primary school | 72%  28% | 44% 19-35 50% 36-50 6% 51-65 | 61% married or de facto with children 6% married or de facto with no children 22% single 11% divorced with children | 33%  60%  40% | 61% have more than one child |
|  | **Thread Clipper** | - Clipping extra  threads from the finished product | - No skill needed | 100% permanent  100% overwork | 57% secondary school 29% other (uneducated) 14% university | 29%  71% | 71% 19-35 29% 36-50 | 100% married or de facto with children | 0% | 86% more than one child |
| RECYCLE | **Logistics Clerk** | - Packing  - Provide assistance in QC and tailoring | - Packing | 100% permanent  100% overwork | 100% other (uneducated) | 0%  100% | 100% 36-50 | 100% married or de facto with children | 0% | 100% less than 3 children |
|  | **Clipper** | - Cutting post-industrial cloth to be sent for making new products  - Arranging the cut cloth  - Clipping extra threads from the finished product | - Cutting | 100% permanent  100% overwork | 40% primary school 40% secondary school 20% technical degree | 100%  0% | 80% 19-35 20% 36-50 | 80% married or de facto with children 20% married or de facto with no children | 0% | 60% more than one child |
